# Supplementary material for: Diversity of platinum-sites at platinum/fullerene interface accelerates alkaline hydrogen evolution
Source: Nat Commun. 2023 Mar 27;14:1711. doi: 10.1038/s41467-023-37404-0 (PMC10042996; doi:10.1038/s41467-023-37404-0)
Supplement: Supplementary file 1 — Supplementary Information [file 41467_2023_37404_MOESM1_ESM.pdf]

Supplementary Information

Diversity of Platinum-sites at Platinum/Fullerene Interface Accelerates Alkaline Hydrogen Evolution

Jiayi Chen[1,‖], Mohammed Aliasgar[1,‖], Fernando Buendia Zamudio[1], Zhang Tianyu[1], Yilin Zhao[1], Xu Lian[2], Lan Wen[1], Haozhou Yang[1], Wenping Sun[3,*], Sergey M. Kozlov[1,*], Wei Chen[2,4,5,6], Lei Wang[1,6*]

[1] Department of Chemical and Biomolecular Engineering, National University of Singapore, 4 Engineering Drive 4, Singapore

[2] Department of Chemistry, National University of Singapore, 3 Science Drive 3, Singapore

[3] School of Materials Science and Engineering, Zhejiang University, Hangzhou, China

[4] Department of Physics, National University of Singapore, 2 Science Drive 3, Singapore, Singapore

[5] Joint School of National University of Singapore and Tianjin University, International Campus of Tianjin University, Binhai New City, Fuzhou, China

[6] Centre for Hydrogen Innovations, National University of Singapore, 1 Engineering Drive 3, Singapore

[‖] These authors contribute to the work equally

E-mail: wenpingsun@zju.edu.cn; sergey.kozlov@nus.edu.sg; wanglei8@nus.edu.sg

Table of Contents

Supplementary Tables 5

Supplementary Table 1. EXAFS fitting parameters at the Pt L_3-edge for various samples (S_0^2=0.85) 5

Supplementary Table 2. The experiment data of exchange currents (mA cm^{-2}) of PtC_{60}, Pt/C, and Pt NCs from the LSV curves used for mass diffusion simulation 6

Supplementary Table 3. ECSA evaluated by the HUPD region of PtC_{60}, Pt/C, Pt NCs, and Pt/CB in 0.1 M KOH 7

Supplementary Table 4. EIS Nyquist plots fitting data of PtC_{60}, Pt/C, and Pt NCs using the equivalent electric circuit in Fig. 3f 8

Supplementary Table 5. DFT (ΔE) and Gibbs free (ΔG) binding energies of H, H_2O, and OH on various atop Pt sites on Pt/C_{60} (011) and Pt/graphene interface, on different positions on unsupported Pt NCs, and on Pt (111) surface. 9

Supplementary Table 6. DFT (ΔE) and Gibbs free (ΔG) binding energies of H_2O, OH, and H on bridge Pt sites on Pt/C_{60}(011) interface, Pt/graphene on unsupported Pt NCs, and Pt (111) surface. 10

Supplementary Table 7. ΔG_R (U = 0 vs RHE) of the HER reaction steps for atop position on the Pt atoms on the interface of Pt/C_{60}(011), Pt/graphene interface, unsupported Pt NCs, and Pt (111) surface. 11

Supplementary Table 8. ΔG_R (U = 0 vs RHE) of the HER reaction steps for bridge Pt sites on the interface of Pt/C_{60} (011), Pt/graphene, as well as on unsupported Pt NCs, and Pt (111) surface. 12

Supplementary Table 9. Activation Energy (E_A) and reaction rate constant (k) at 0 V vs RHE for the various forward alkaline HER reaction steps for atop sites on the Pt atoms on the interface of Pt/C_{60}(011), Pt/graphene interface, various positions on unsupported Pt NCs, and Pt (111) surface. 13

Supplementary Table 10. Activation Energy (E_A) and reaction rate constant (k) at 0 V vs RHE for the various forward alkaline HER reaction steps for bridge sites on the Pt atoms on the interfaces of Pt/C_{60}(011), Pt/graphene as well as different bridge sites on unsupported Pt NC, and Pt (111) surface. 14

Supplementary Table 11. The power and efficiency of the AEM electrolyser employing PtC_{60} and reference cathode catalysts operated at 1 A cm^{-2} 15

Supplementary Figures 16

Supplementary Fig. 1. Structure of C_{60} bulk crystal measured by TEM and AFM 16

Supplementary Fig. 2. XRD analysis of the PtC_{60}, C_{60} crystal bulk, and Pt NCs 17

Supplementary Fig. 3. Structure of PtC_{60} measured by AFM 18

Supplementary Fig. 4. Nanostructure of PtC_{60} measured by TEM 19

Supplementary Fig. 5. Tyndall effect of PtC_{60} 20

Supplementary Fig. 6. Function groups on PtC_{60} analysed by FT-IR 21

Supplementary Fig. 7. TEM images and size distribution of Pt nanoclusters in PtC_{60}-32.5 wt% 22

Supplementary Fig. 8. TEM images and size distribution of Pt nanoclusters in PtC_{60}-47.5 wt% 23

Supplementary Fig. 9. TEM images of PtC_{60} after calcination 24

Supplementary Fig. 10. TEM images of Pt/C before and after calcination............................25
Supplementary Fig. 11. TEM images of Pt/CB before and after calcination..........................26
Supplementary Fig. 12. TEM images of Pt NCs...27
Supplementary Fig. 13. STEM image and HAADF image of PtC_{60}..28
Supplementary Fig. 14. XPS survey spectra of PtC_{60}, Pt NCs, and C_{60} bulk crystal..............29
Supplementary Fig. 15. XPS O *1s* spectra of PtC_{60}, Pt NCs and C_{60} precursor powder........30
Supplementary Fig. 16. XPS of Pt *4f* of Pt/CB and Pt/C 31
Supplementary Fig. 17. HER activity and HUPD of Pt/CB with various catalyst loading.....32
Supplementary Fig. 18. Schematic illustration of XPS/UPS measurement............................34
Supplementary Fig. 19. EXAFS spectra and fitting data for PtC_{60} and Pt NCs......................35
Supplementary Fig. 20. Wavelet transform for the k^3-weighted EXAFS spectra of Pt foil and PtO_2..36
Supplementary Fig. 21. HER activity of PtC_{60} with various Pt loading in 1.0 M KOH.........37
Supplementary Fig. 22. Stability performance of PtC_{60} and reference samples.....................38
Supplementary Fig. 23. TEM of PtC_{60} and size distribution of Pt nanoclusters post-test.......39
Supplementary Fig. 24. Hydrophilicity of PtC_{60}, C_{60} bulk crystal and Pt NCs.......................40
Supplementary Fig. 25. Tafel plot of PtC_{60} and reference samples..41
Supplementary Fig. 26. Simulation of HER current-potential curves limited by the inefficient mass transportation 42
Supplementary Fig. 27. HER activity of PtC_{60} and reference samples with low-catalyst loadings in 1.0 M KOH normalized by geometry area 43
Supplementary Fig. 28. HER activity of PtC_{60} and reference samples with various catalyst loadings in 1.0 M KOH normalized by Pt mass loading...44
Supplementary Fig. 29. HUPD (hydrogen underpotential deposition) of PtC_{60} and reference samples suggested by cyclic voltammetry curves..45
Supplementary Fig. 30. HER activity of PtC_{60} and reference samples with various catalyst loadings in 1.0 M KOH normalized by ECSA..46
Supplementary Fig. 31. Tafel plot of PtC_{60} and reference samples with different catalyst loadings normalized by electrode geometry area in 1.0 M KOH..47
Supplementary Fig. 32. HER activity of PtC_{60} and reference samples with various catalyst loading in 0.5 M H_2SO_4 normalized by geometry area...48
Supplementary Fig. 33. Nyquist plots of PtC_{60}, Pt/C, and Pt NCs in 1.0 M KOH................49
Supplementary Fig. 34. Correlation between MEAM and DFT binding energies.................50
Supplementary Fig. 35. Bader charge on Pt atoms in Pt/C_{60} model..51
Supplementary Fig. 36. Calculated work functions of Pt NCs 52
Supplementary Fig. 37. Density of states (DOS) per atom for the carbon atoms. 53
Supplementary Fig. 38. Pt NC adsorbed on C_{60}(011) and Pt/graphene systems. 54
Supplementary Fig. 39. Color Map to illustrate reaction rate constant on different atop Pt sites for Pt/C_{60} (011) for different forward alkaline HER reaction steps..55
Supplementary Fig. 40. Gibbs free energy profile for H diffusion 56
Supplementary Fig. 41. Simulated LSV for 17 Pt surface interfacial sites on Pt/C_{60} (011), 16 Pt surface interfacial sites on Pt/graphene, and unsupported Pt NCs.....................................57
Supplementary Fig. 42. Photograph of the AEM electrolyser 58
Supplementary Fig. 43. Fitting procedure of UPS spectra during Pt deposition on C_{60} film..59

Supplementary Notes..**60**
Supplementary Note 1. Effect of hydrophilicity on catalytic stability....................................60
Supplementary Note 2. Mass diffusion effect..61
Supplementary Note 3. Design and optimization of the calculated catalyst model................62
Supplementary Note 4. DFT optimization of Pt/C_{60} structures..65
Supplementary Note 5. Analysis of the electron transfer through the Pt/C_{60}(011) interface...66
Supplementary Note 6. The work function of the considered Pt/C_{60} model 67
Supplementary Note 7. Molecular adsorption..68
Supplementary Note 8. Reaction mechanism 70
Supplementary Note 9. Efficiency of AEM electrolyser:...75
References..**77**

Supplementary Tables

Supplementary Table 1. EXAFS fitting parameters at the Pt L_3-edge for various samples (S_0^2=0.85)

	shell	*CN*	R(Å)	σ^2	ΔE^0	*R* factor
Pt foil	Pt-Pt	12	2.76±0.01	0.0050	7.2±0.5	0.0031
PtC_{60}	Pt-O	0.5±0.2	2.04±0.04	0.0059	9.2±1.0	0.0067
	Pt-Pt	7.9±0.3	2.76±0.01	0.0058		
Pt NCs	Pt-O	0.9±0.3	1.92±0.02	0.0019	10.8±2.1	0.0145
	Pt-O1	0.9±0.4	2.17±0.03	0.0019		
	Pt-Pt	8.1±0.6	2.77±0.01	0.0054		

CN: coordination numbers; *R*: bond distance; σ^2: Debye-Waller factors; ΔE_0: the inner potential correction. *R* factor: goodness of fit. S_0^2 was set to 0.85, according to the experimental EXAFS fit of Pt foil reference by fixing CN as the known crystallographic value.

The quantitative analysis results indicated the lower coordination number of Pt-Pt bonding in PtC_{60} than that of Pt NCs, which can be attributed to the less aggregation of Pt nanoclusters on C_{60} compared to that of Pt NCs.

Supplementary Table 2. The experiment data of exchange currents (mA cm^{-2}) of PtC_{60}, Pt/C, and Pt NCs from the LSV curves used for mass diffusion simulation

Loading of samples	0.4 mg cm^{-2}	0.04 mg cm^{-2}	0.004 mg cm^{-2}
PtC_{60}	1.766	0.446	0.0368
Pt/C	0.668	0.0783	0.00186
Pt NCs	0.614	0.0313	0.00114

Supplementary Table 3. ECSA evaluated by the HUPD region of PtC_{60}, Pt/C, Pt NCs, and Pt/CB in 0.1 M KOH

Loading amounts	0.4 mg cm^{-2} (50μg on RDE)	0.04 mg cm^{-2} (5μg on RDE)	0.004 mg cm^{-2} (0.5μg on RDE)
PtC_{60}	5.08 cm^2	0.45 cm^2	0.039 cm^2
Pt/C	5.00 cm^2	0.480 cm^2	0.037 cm^2
Pt NCs	4.42 cm^2	0.42 cm^2	0.044 cm^2
Pt/CB	10.32 cm^2	2.56 cm^2	0.82 cm^2

Supplementary Table 4. EIS Nyquist plots fitting data of PtC_{60}, Pt/C, and Pt NCs using the equivalent electric circuit in Fig. 3f

Catalysts	PtC_{60}	Pt/C	Pt NCs
R_S (ohm)	0.81	0.9	0.9
R_{CT} (ohm)	4	96	102
CPE_{DL} (F s^(α-1) cm^{-2})	1.5E–3	9E–6	1.1E–5
A_{DL}	0.9	0.795	0.795
R_P (ohm)	41	73	140
CPE_P (F s^(α-1) cm^{-2})	5.5E–3	7.4E–5	5.5E–5
A_P	0.774	0.69	0.69

In a typical measurement, two semicircles were observed for all three electrocatalysts. As suggested previously,[1-3] the first semicircle at high frequencies reveals the electron transfer kinetics induced by the adsorption of surface H, which is associated to the charge transfer resistance (R_{CT}) and the double layer capacitance (constant phase elements, CPE_{DL}). The second semicircle at low frequencies region is a result of H coverage modulation by electrode-potential oscillations, corresponding to a pseudocapacitive behavior (adsorption resistance (R_P) and pseudocapacitance (CPE_P)). A_{DL} and $_{AP}$ are the coefficients of CPE_{DL} and CPE_P, respectively. R_S represents the solution resistance.

Supplementary Table 5. DFT (ΔE) and Gibbs free (ΔG) binding energies of H, H_2O, and OH on various atop Pt sites on Pt/C_{60} (011) and Pt/graphene interface, on different positions on unsupported Pt NCs, and on Pt (111) surface.

	H binding		H_2O adsorption		OH binding	
**Position**	E_{ads} (eV)	G_{ads} (eV)	E_{ads} (eV)	G_{ads} (eV)	E_{ads} (eV)	G_{ads} (eV)
**1**	–0.47	–0.40	–0.65	–0.22	0.34	0.76
**2**	–0.29	–0.20	–0.60	–0.01	0.29	0.67
**3**	–0.33	–0.24	–0.43	0.09	0.16	0.58
**4**	–0.37	–0.31	–0.18	0.43	0.14	0.47
**5**	–0.46	–0.39	–0.49	–0.08	0.29	0.69
**6**	–0.47	–0.39	–1.02	–0.46	0.03	0.40
**7**	–0.71	–0.62	–0.68	–0.10	0.24	0.57
**8**	–0.44	–0.35	–0.57	0.02	0.12	0.52
**9**	–0.27	–0.17	–0.53	0.05	0.10	0.50
**10**	–0.48	–0.41	–0.69	–0.14	0.10	0.50
**11**	–0.54	–0.47	–0.74	–0.16	–0.05	0.34
**12**	–0.32	–0.25	–0.63	–0.09	0.16	0.57
**13**	–0.45	–0.38	–0.74	–0.15	0.41	0.84
**14**	–0.51	–0.42	–0.79	–0.22	0.37	0.75
**15**	–0.49	–0.41	–0.54	0.01	0.55	0.93
**16**	–0.54	–0.45	–0.07	0.31	0.30	0.65
**17**	–0.66	–0.59	–0.63	–0.08	–0.30	0.12
**Pt/graphene Corner**	–0.58	–0.45	–0.30	–0.04	–0.01	0.25
**Pt/graphene Edge**	–0.46	–0.26	–0.32	0.38	0.47	0.74
**Unsupported Pt NCs Corner**	–0.54	–0.46	–0.63	–0.02	–0.13	0.27
**Unsupported Pt NCs Edge**	–0.36	–0.29	–0.50	0.36	0.33	0.73
**Unsupported Pt NCs**	–0.46	–0.43	–0.50	0.10	0.68	1.05
**Pt (111)**	-0.49	-0.29	-0.47	0.08	0.66	1.01

Supplementary Table 6. DFT (ΔE) and Gibbs free (ΔG) binding energies of H_2O, OH, and H on bridge Pt sites on Pt/C_{60}(011) interface, Pt/graphene on unsupported Pt NCs, and Pt (111) surface.

	H_2O adsorption		H adsorption		OH adsorption	
**Binding Position**	E_{ads} (eV)	G_{ads} (eV)	E_{ads} (eV)	G_{ads} (eV)	E_{ads} (eV)	G_{ads} (eV)
**1-2**	–0.63	–0.08	–0.47	–0.27	0.04	0.42
**1-3**	–0.63	–0.09	–0.42	–0.24	0.23	0.63
**2-4**	–0.66	–0.11	–0.39	–0.24	0.93	1.40
**2-8**	–0.65	–0.22	–0.55	–0.34	0.41	0.86
**2-9**	–0.68	–0.10	–0.52	–0.32	0.32	0.77
**3-4**	–0.67	–0.11	–0.41	–0.28	–0.19	0.16
**3-15**	–1.02	–0.46	–0.47	–0.26	0.21	0.64
**4-5**	–0.69	–0.14	–0.56	–0.40	–0.09	0.26
**5-6**	–0.57	0.02	–0.66	–0.50	0.68	1.09
**5-8**	–0.43	0.09	0.01	0.21	1.01	1.44
**6-7**	–0.49	–0.08	–0.49	–0.31	–0.13	0.27
**7-8**	–0.07	0.31	–0.86	–0.66	0.08	0.48
**8-9**	–0.60	–0.01	–0.57	–0.36	0.02	0.42
**9-10**	–0.79	–0.22	–0.62	–0.41	0.14	0.53
**11-12**	–0.54	0.01	–0.65	–0.47	0.29	0.69
**13-14**	–0.18	0.43	–0.73	–0.54	0.24	0.59
**13-16**	–0.54	0.05	–0.52	–0.35	1.34	1.79
**14-17**	–0.53	0.05	–0.20	–0.03	–0.24	0.18
**15-16**	–0.53	0.00	–0.79	–0.59	–0.15	0.27
**16-17**	–0.74	–0.16	–0.92	–0.73	–0.57	–0.15
**Pt/graphene Corner - Edge**	-0.36	0.19	-0.62	-0.42	0.93	1.40
**Unsupported Pt NCs Corner – Corner**	–0.63	–0.02	–0.64	–0.46	0.23	0.63
**Unsupported Pt NCs Corner - Edge**	–0.50	0.36	–0.69	–0.46	0.05	0.46
**Unsupported Pt NCs Terrace-Terrace**	–0.50	0.10	–0.49	–0.32	0.24	0.59
**Pt (111) Bridge Position**	-0.55	0.12	-0.47	-0.38	0.18	0.72

Supplementary Table 7. ΔG$_R$ (U = 0 vs RHE) of the HER reaction steps for atop position on the Pt atoms on the interface of Pt/C$_{60}$(011), Pt/graphene interface, unsupported Pt NCs, and Pt (111) surface.

G$_R$ (U = 0 vs RHE) (eV)				
**Binding Position**	**Volmer**	**Heyrovsky**	**Tafel**	**Chemical Water Dissociation**
**1**	–0.40	0.40	0.40	0.36
**2**	–0.20	0.20	0.20	0.47
**3**	–0.24	0.24	0.24	0.34
**4**	–0.31	0.31	0.31	0.16
**5**	–0.39	0.39	0.39	0.30
**6**	–0.39	0.39	0.39	0.01
**7**	–0.62	0.62	0.62	–0.05
**8**	–0.35	0.35	0.35	0.17
**9**	–0.17	0.17	0.17	0.33
**10**	–0.41	0.41	0.41	0.09
**11**	–0.47	0.47	0.47	–0.13
**12**	–0.25	0.25	0.25	0.33
**13**	–0.38	0.38	0.38	0.46
**14**	–0.42	0.42	0.42	0.32
**15**	–0.41	0.41	0.41	0.52
**16**	–0.45	0.45	0.45	0.20
**17**	–0.59	0.59	0.59	–0.46
**Pt/graphene Corner**	–0.45	0.45	0.45	–0.20
**Pt/graphene Edge**	–0.26	0.26	0.26	0.48
**Unsupported Pt NCs Corner**	–0.46	0.46	0.46	–0.19
**Unsupported Pt NCs Edge**	–0.30	0.30	0.30	0.44
**Unsupported Pt NCs Terrace**	–0.33	0.33	0.33	0.72
**Pt(111)**	–0.29	0.29	0.29	0.72

Supplementary Table 8. ΔG_R (U = 0 vs RHE) of the HER reaction steps for bridge Pt sites on the interface of Pt/C_{60} (011), Pt/graphene, as well as on unsupported Pt NCs, and Pt (111) surface.

G_R (U = 0 vs RHE) (eV)				
**Binding Position**	**Volmer**	**Heyrovsky**	**Tafel**	**Chemical Water Dissociation**
**1-2**	–0.21	0.21	0.21	0.85
**1-3**	–0.40	0.40	0.40	0.40
**2-4**	–0.26	0.26	0.26	1.14
**2-8**	–0.24	0.24	0.24	0.61
**2-9**	–0.34	0.34	0.34	0.42
**3-4**	–0.35	0.35	0.35	0.35
**3-15**	–0.28	0.28	0.28	0.37
**4-5**	–0.50	0.50	0.50	0.50
**5-6**	–0.24	0.24	0.24	0.85
**5-8**	–0.27	0.27	0.27	2.17
**6-7**	–0.32	0.32	0.32	0.32
**7-8**	–0.47	0.47	0.47	0.47
**8-9**	–0.59	0.59	0.59	0.59
**9-10**	–0.41	0.41	0.41	0.41
**11-12**	–0.31	0.31	0.31	0.38
**13-14**	–0.73	0.73	0.73	0.73
**13-16**	–0.03	0.03	0.03	4.76
**14-17**	–0.66	0.66	0.66	0.66
**15-16**	–0.54	0.54	0.54	0.54
**16-17**	–0.36	0.36	0.36	0.36
**Pt/graphene Corner - Edge**	-0.42	0.42	0.42	1.40
**Unsupported Pt NC Corner**	–0.46	0.46	0.46	0.46
**Unsupported Pt NC Edge**	–0.46	0.46	0.46	0.46
**Unsupported Pt NC Terrace**	–0.32	0.32	0.32	0.88
**Pt(111)**	–0.25	0.25	0.25	0.74

Supplementary Table 9. Activation Energy (E_A) and reaction rate constant (k) at 0 V vs RHE for the various forward alkaline HER reaction steps for atop sites on the Pt atoms on the interface of Pt/C_{60}(011), Pt/graphene interface, various positions on unsupported Pt NCs, and Pt (111) surface.

Binding Position	Alkaline Volmer		Alkaline Heyrovsky		Tafel		Chemical Water Dissociation	
	E_A (eV)	k (s^{-1})	E_A (eV)	k (s^{-1})	E_A (eV)	k (s^{-1})	E_A (eV)	k (s^{-1})
**1**	0.30	4.50E+07	1.32	3.54E–10	0.60	3.82E+02	0.66	4.31E+01
**2**	0.51	1.80E+04	1.11	8.85E–07	0.81	1.50E–01	0.77	5.70E–01
**3**	0.47	6.77E+04	1.15	2.36E–07	0.77	5.70E–01	0.64	9.39E+01
**4**	0.40	1.16E+06	1.22	1.38E–08	0.70	9.85E+00	0.46	1.21E+05
**5**	0.32	2.51E+07	1.30	6.35E–10	0.62	2.13E+02	0.60	3.96E+02
**6**	0.32	2.61E+07	1.30	6.11E–10	0.62	2.22E+02	0.31	3.42E+07
**7**	0.09	2.27E+11	1.53	7.04E–14	0.39	1.92E+06	0.25	3.82E+08
**8**	0.36	4.53E+06	1.26	3.52E–09	0.66	3.85E+01	0.47	6.75E+04
**9**	0.54	5.19E+03	1.08	3.08E–06	0.84	4.00E–02	0.63	1.62E+02
**10**	0.30	5.47E+07	1.32	2.92E–10	0.60	4.64E+02	0.39	1.64E+06
**11**	0.24	5.65E+08	1.38	2.82E–11	0.54	4.80E+03	0.17	7.95E+09
**12**	0.46	9.61E+04	1.16	1.66E–07	0.76	8.20E–01	0.63	1.56E+02
**13**	0.33	1.99E+07	1.29	8.03E–10	0.63	1.69E+02	0.76	1.03E+00
**14**	0.29	9.43E+07	1.33	1.69E–10	0.59	8.01E+02	0.63	1.68E+02
**15**	0.30	5.47E+07	1.32	2.92E–10	0.60	4.64E+02	0.83	7.00E–02
**16**	0.26	2.80E+08	1.36	5.69E–11	0.56	2.38E+03	0.50	2.02E+04
**17**	0.12	5.16E+10	1.50	3.09E–13	0.42	4.38E+05	0	6.21E+12
**Pt/graphene Corner**	0.26	1.05E+09	1.36	2.44E–10	0.56	1.55E+04	0.10	8.61E+11
**Pt/graphene Edge**	0.45	6.52E+05	1.17	3.92E–07	0.75	8.30E+00	0.78	2.85E+00
**Unsupported Pt NC Corner**	0.25	2.96E+09	1.37	2.64E–10	0.55	2.51E+04	0.11	7.00E+11
**Unsupported Pt NC Edge**	0.42	3.14E+06	1.20	1.83E–07	0.72	2.66E+01	0.74	1.15E+01
**Unsupported Pt NC Terrace**	0.38	9.92E+06	1.24	2.57E–08	0.68	8.42E+01	1.02	1.20E–04
**Pt (111)**	0.71	6.47E+00	0.91	2.47E-03	1.01	5.49E-05	0.30	8.24E-06

Supplementary Table 10. Activation Energy (E_A) and reaction rate constant (k) at 0 V vs RHE for the various forward alkaline HER reaction steps for bridge sites on the Pt atoms on the interfaces of Pt/C_{60}(011), Pt/graphene as well as different bridge sites on unsupported Pt NC, and Pt (111) surface.

Binding Position	Volmer		Heyrovsky		Tafel		Chemical Water Dissociation	
	E_A (eV)	k (s^{-1})	E_A (eV)	k (s^{-1})	E_A (eV)	k (s^{-1})	E_A (eV)	k (s^{-1})
**1-2**	0.92	2.12E–03	0.70	7.54E+00	1.22	1.80E–08	1.15	2.34E–07
**1-3**	0.31	3.56E+07	1.31	4.48E–10	0.61	3.03E+02	0.53	6.04E+03
**2-4**	0.44	1.94E+05	1.18	8.24E–08	0.74	1.64E+00	1.44	3.04E–12
**2-8**	0.46	8.89E+04	1.16	1.79E–07	0.76	7.55E–01	0.91	2.28E–03
**2-9**	0.36	4.36E+06	1.26	3.66E–09	0.66	3.70E+01	0.72	4.01E+00
**3-4**	0.36	5.09E+06	1.26	3.13E–09	0.66	4.32E+01	0.11	8.21E+10
**3-15**	0.43	3.09E+05	1.19	5.17E–08	0.73	2.62E+00	0.67	3.41E+01
**4-5**	0.21	1.62E+09	1.41	9.87E–12	0.51	1.37E+04	0.07	4.38E+11
**5-6**	0.47	7.32E+04	1.15	2.18E–07	0.77	6.21E–01	1.15	2.25E–07
**5-8**	0.44	2.18E+05	1.18	7.33E–08	0.74	1.85E+00	2.47	9.37E–30
**6-7**	0.38	2.00E+06	1.24	7.98E–09	0.68	1.70E+01	0.25	4.13E+08
**7-8**	0.24	5.43E+08	1.38	2.94E–11	0.54	4.61E+03	0.31	3.70E+07
**8-9**	0.12	6.03E+10	1.50	2.64E–13	0.42	5.12E+05	0.13	4.08E+10
**9-10**	0.30	6.39E+07	1.32	2.50E–10	0.60	5.43E+02	0.42	4.73E+05
**11-12**	0.40	1.21E+06	1.22	1.32E–08	0.70	1.02E+01	0.68	1.98E+01
**13-14**	0.00	1.70E+13	1.65	9.36E–16	0.27	1.45E+08	0.15	1.60E+10
**13-16**	0.68	2.06E+01	0.94	7.73E–04	0.98	1.75E–04	5.06	1.75E–73
**14-17**	0.05	9.20E+11	1.57	1.73E–14	0.35	7.81E+06	0.00	6.21E+12
**15-16**	0.17	8.29E+09	1.45	1.92E–12	0.47	7.04E+04	0.03	2.08E+12
**16-17**	0.35	8.44E+06	1.27	1.89E–09	0.65	7.17E+01	0.00	6.21E+12
**Pt/graphene Corner - Edge**	0.29	4.33E+13	1.33	1.92E-12	0.59	5.30E+03	1.51	1.07E-12
**Unsupported Pt Corner - Corner**	0.25	2.84E+09	1.37	2.75E–10	0.55	2.41E+04	0.12	4.56E+11
**Unsupported Pt Corner - Edge**	0.25	2.09E+09	1.37	2.75E–10	0.55	1.77E+04	0.30	2.91E+08
**Unsupported Pt Terrace-Terrace**	0.38	8.02E+06	1.24	3.18E–08	0.68	6.81E+01	1.18	2.97E–07
**Pt (111) Bridge Position**	0.59	6.06E+02	1.14	3.19E-07	0.92	1.82E-03	0.50	3.43E-09

Supplementary Table 11. The power and efficiency of the AEM electrolyser employing PtC_{60} and reference cathode catalysts operated at 1 A cm^{-2}

Cathode catalysts	Power of AEM electrolyser (W cm^{-2})	Efficiency (%)
PtC_{60}	1.87	79.1
Pt/C	2.21	66.9
Pt NCs	2.50	59.2

Supplementary Figures

Supplementary Fig. 1. Structure of C_{60} bulk crystal measured by TEM and AFM

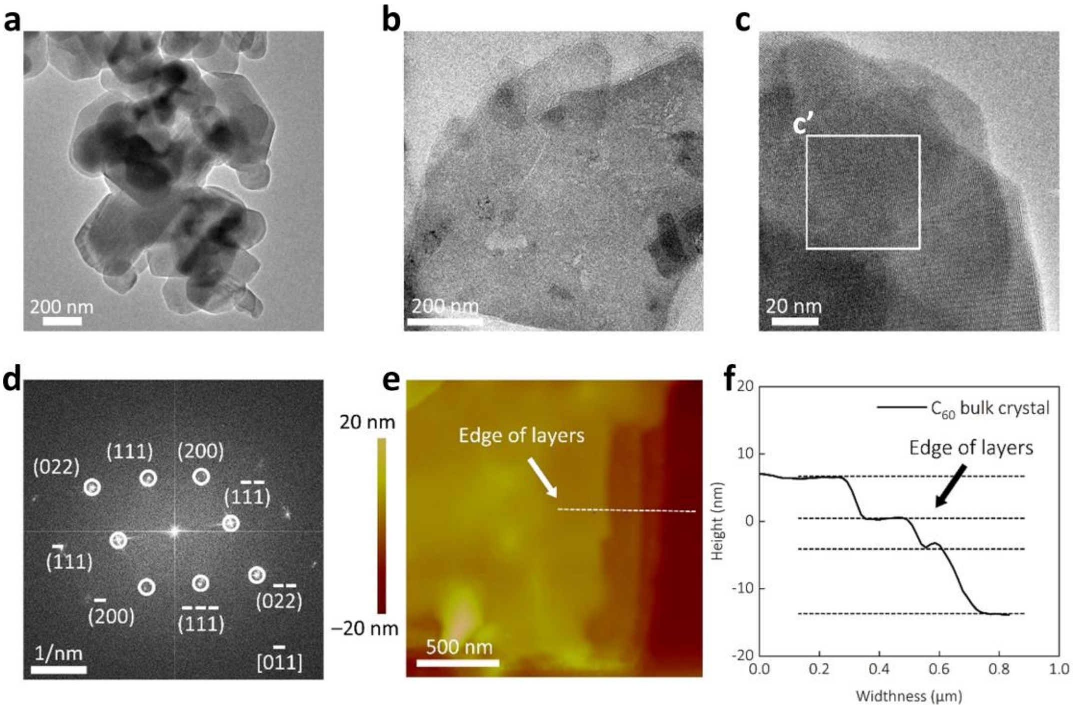

Supplementary Fig. 1. a-c) TEM images of the C_{60} bulk crystal, d) the FFT pattern derived from the selected area **c'** in c), e) typical AFM image of C_{60} bulk crystal, f) depth profile of a cross-section corresponding to the line in the e).

Supplementary Fig. 2. XRD analysis of the PtC_{60}, C_{60} crystal bulk, and Pt NCs

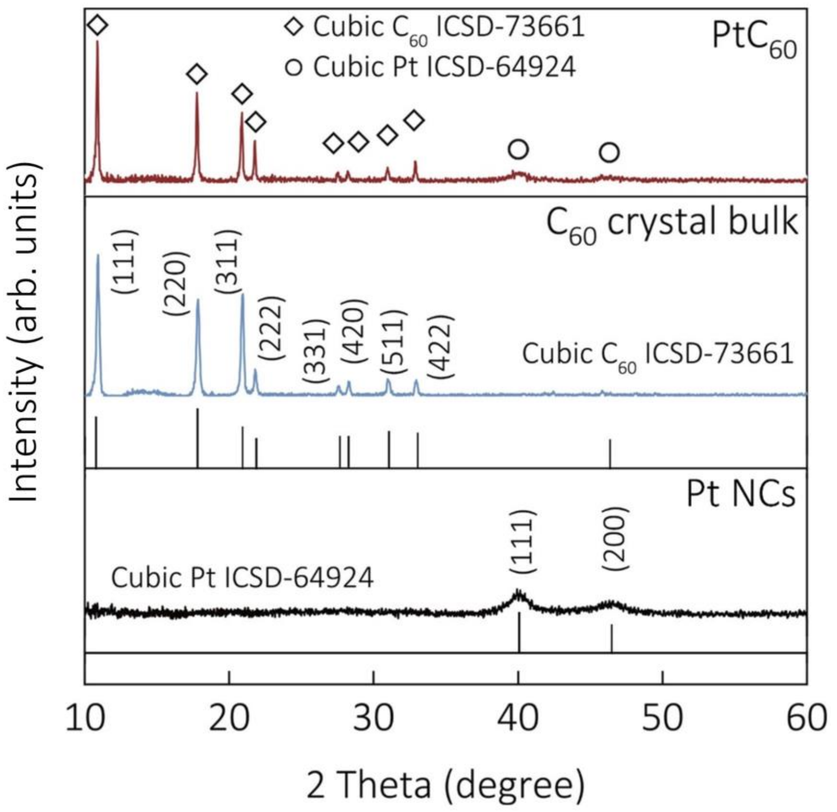

Supplementary Fig. 2. XRD patterns of the PtC_{60}, C_{60} bulk crystal, and Pt NCs.

Supplementary Fig. 3. Structure of PtC_{60} measured by AFM

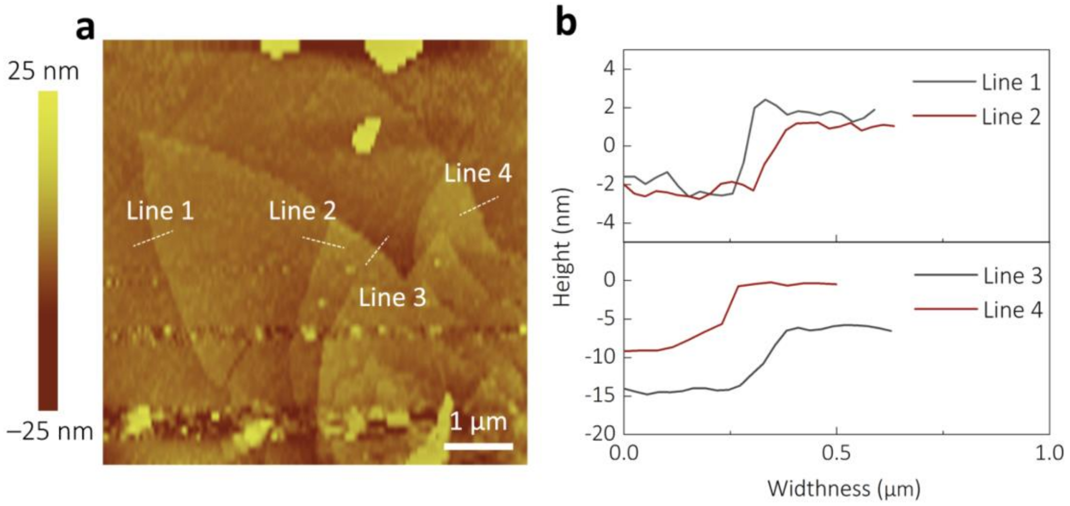

Supplementary Fig. 3. a) AFM image of PtC_{60}, (b) depth profile of a cross-section corresponding to the line in the a)

Supplementary Fig. 4. Nanostructure of PtC_{60} measured by TEM

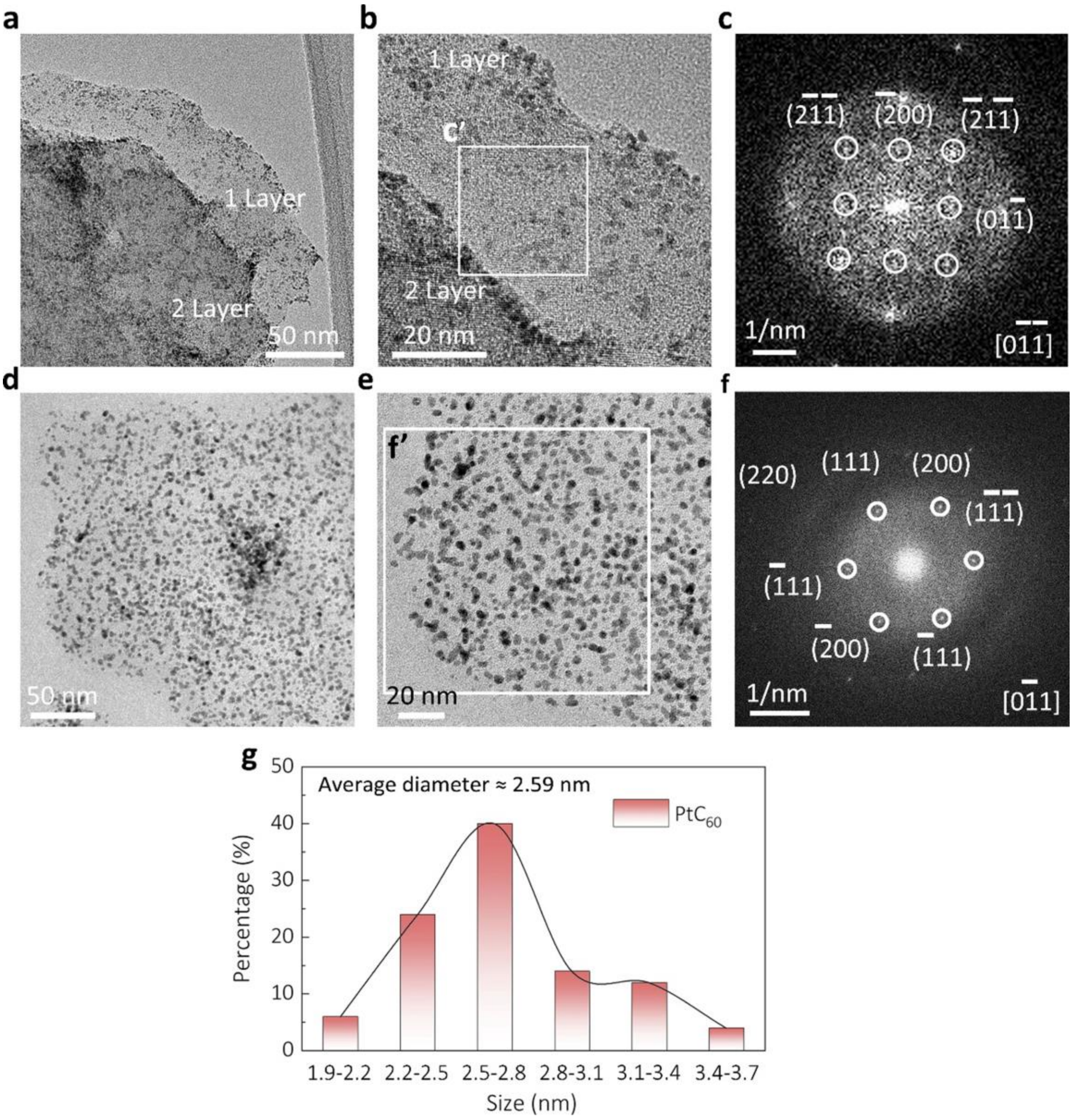

Supplementary Fig. 4. a-b) TEM images of the stacked edges of PtC_{60}, and c) corresponding fast Fourier transform (FFT) pattern derived for the selected area c' in b), displaying the specific lattice plane of C_{60}. d-e) TEM image of PtC_{60}, and f) corresponding fast Fourier transform (FFT) pattern derived from the selected area f' in e), displaying the specific lattice plane of C_{60}. g) the size distribution histogram of Pt nanoclusters in PtC_{60}.

Supplementary Fig. 5. Tyndall effect of PtC_{60}

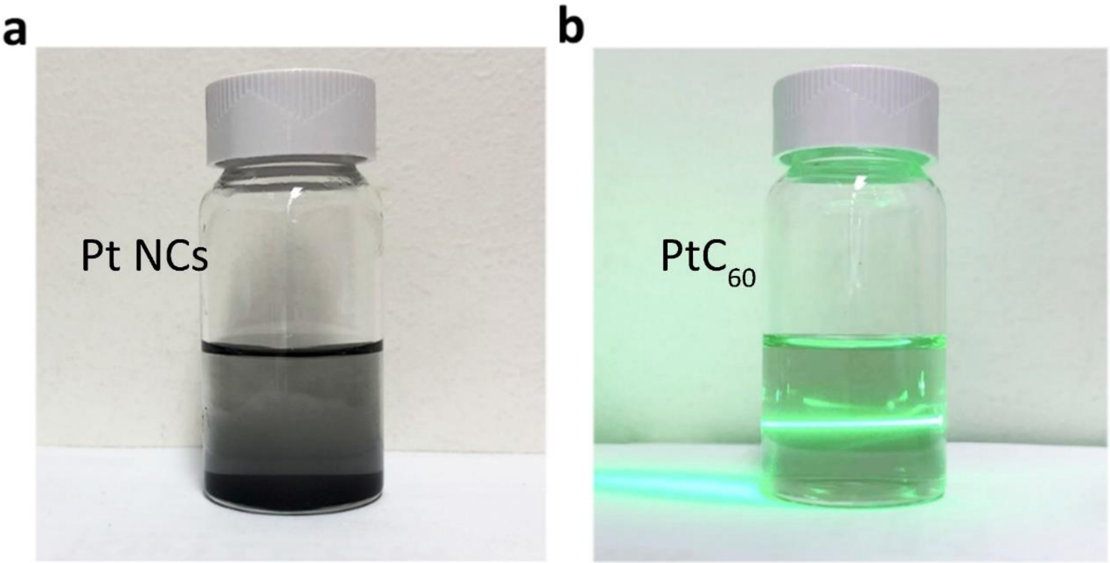

Supplementary Fig. 5. a) optical photo of Pt NCs in ethanol, b) optical photo of the PtC_{60} in ethanol displaying typical Tyndall effect.

Supplementary Fig. 6. Function groups on PtC_{60} analysed by FT-IR

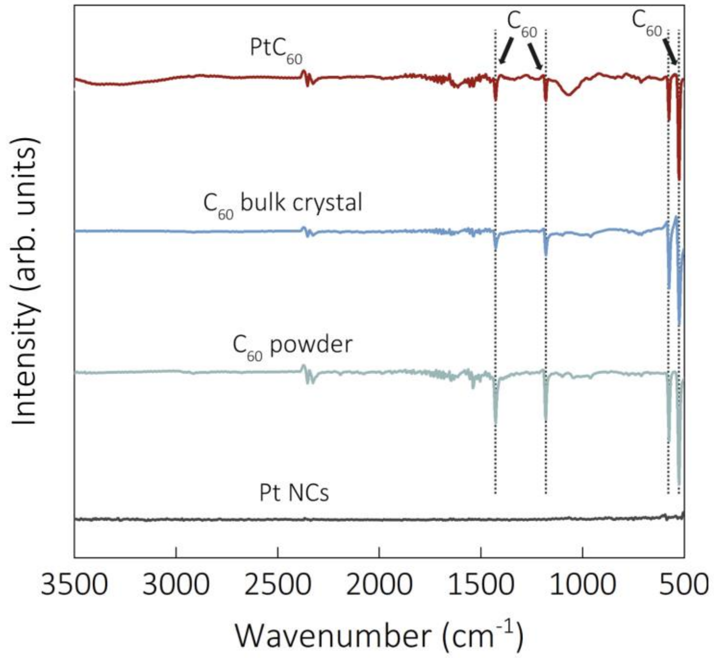

Supplementary Fig. 6. Fourier transform infrared (FT-IR) spectra of PtC_{60}, C_{60} bulk crystal, C_{60} powder, and Pt NCs.

Supplementary Fig. 7. TEM images and size distribution of Pt nanoclusters in PtC_{60}-32.5 wt%

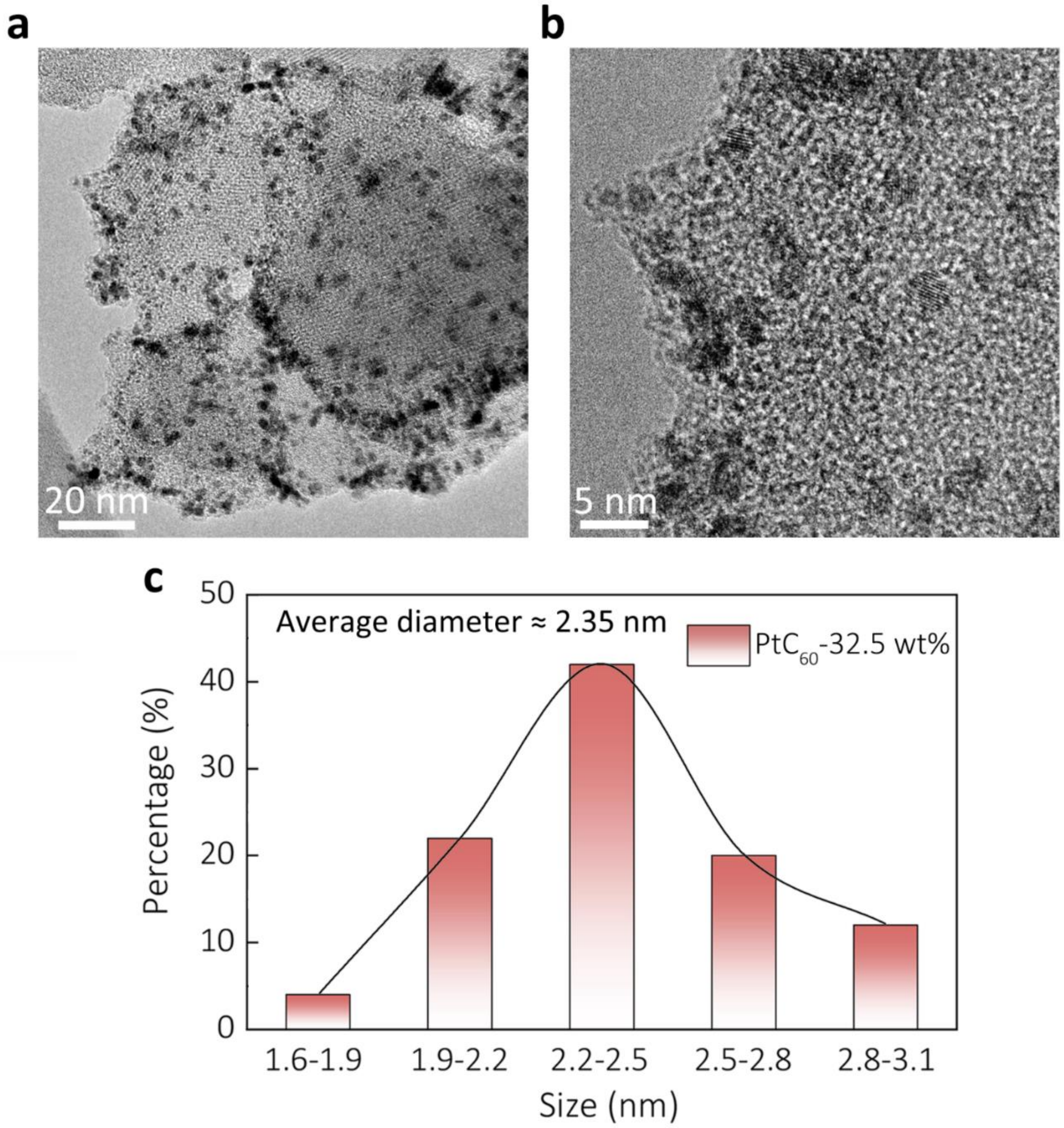

Supplementary Fig. 7. a-b) TEM images of PtC_{60}-32.5 wt%, c) size distribution histogram of Pt nanoclusters in PtC_{60}-32.5 wt%.

Supplementary Fig. 8. TEM images and size distribution of Pt nanoclusters in PtC_{60}-47.5 wt%

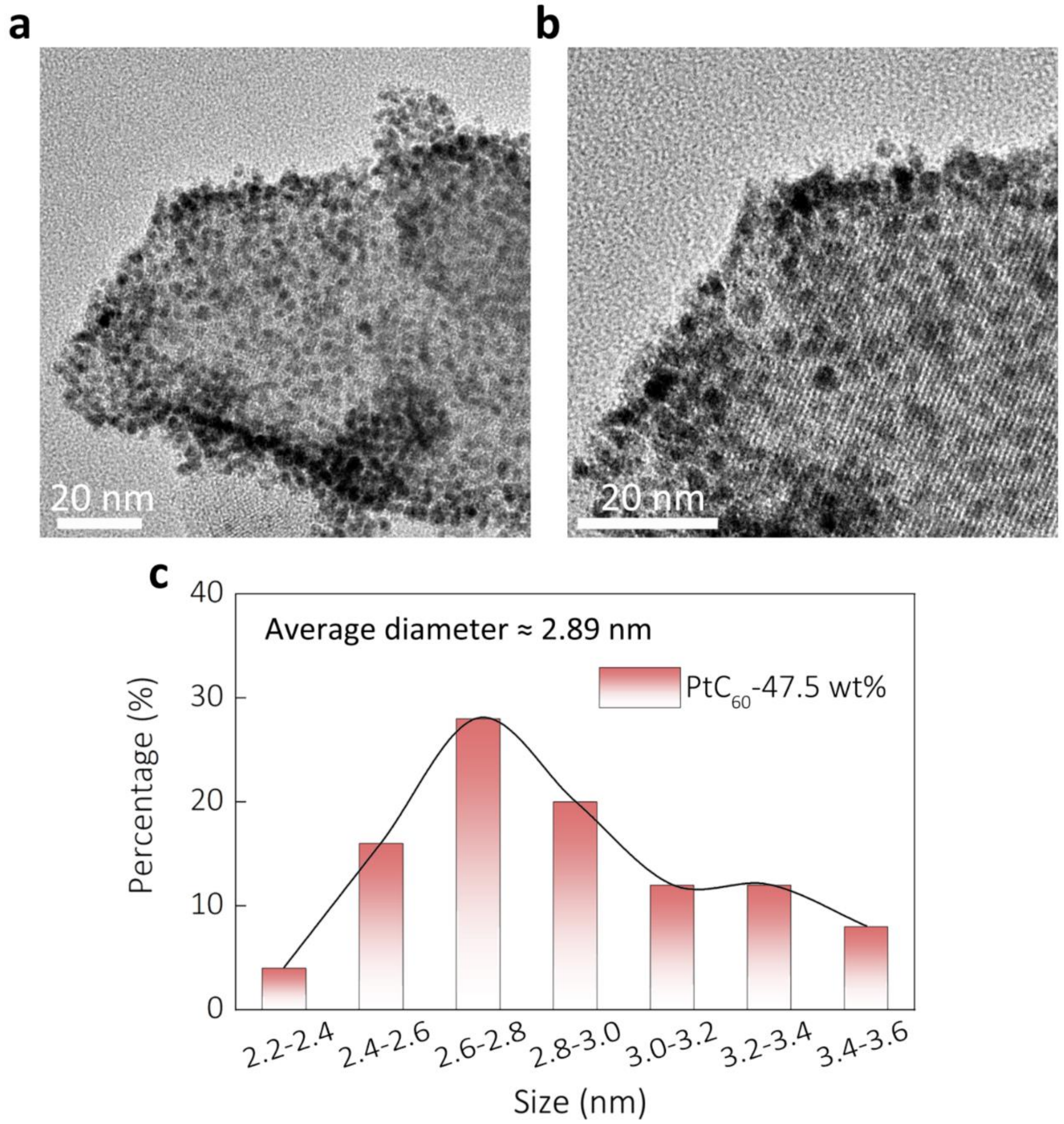

Supplementary Fig. 8. a-b) TEM images of PtC_{60}-47.5 wt%, c) size distribution histogram of Pt nanoclusters in PtC_{60}-47.5 wt%.

Supplementary Fig. 9. TEM images of PtC_{60} after calcination

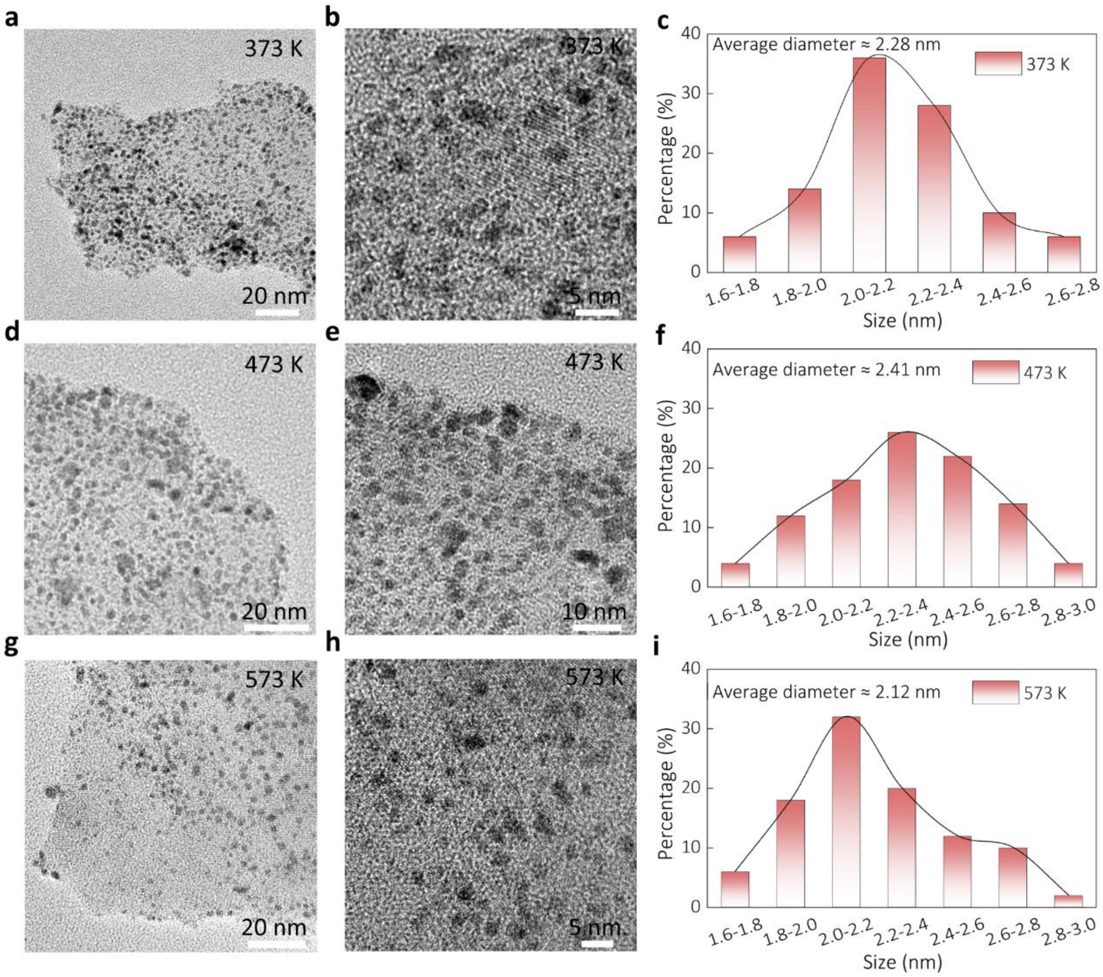

Supplementary Fig. 9. TEM images of PtC_{60} calcinated for 1 hour at a-b) 373 K, d-e) 473 K, and g-h) 573 K, c, f, i) the corresponding size distribution histogram of Pt nanoclusters in PtC_{60}.

Supplementary Fig. 10. TEM images of Pt/C before and after calcination

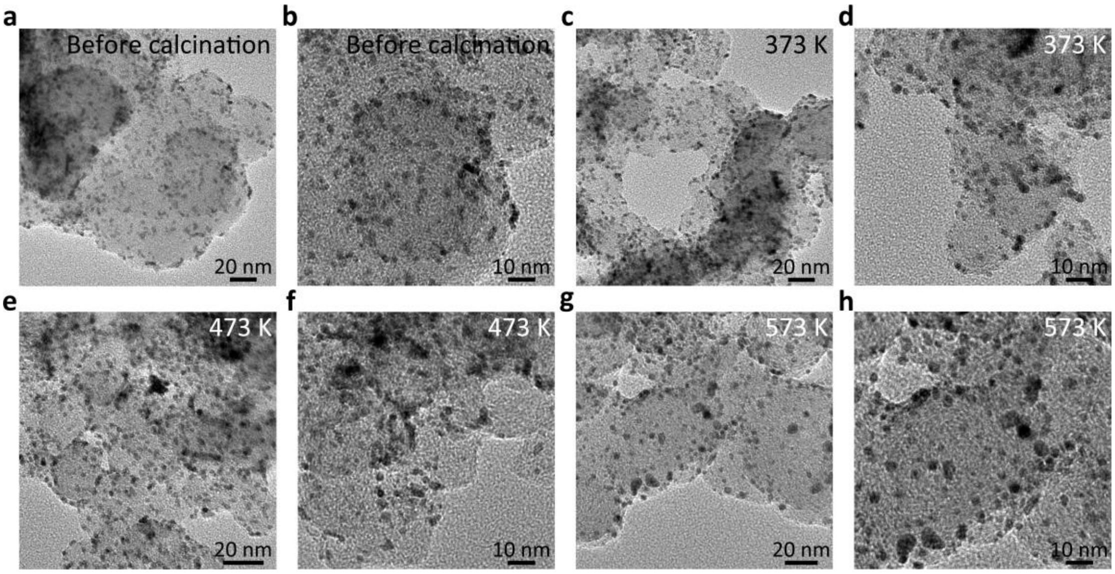

Supplementary Fig. 10. TEM images of commercial Pt/C a) before calcination, and after calcination for 1 hour at c-d) 373 K, e-f) 473 K, and g-h) 573 K.

Supplementary Fig. 11. TEM images of Pt/CB before and after calcination

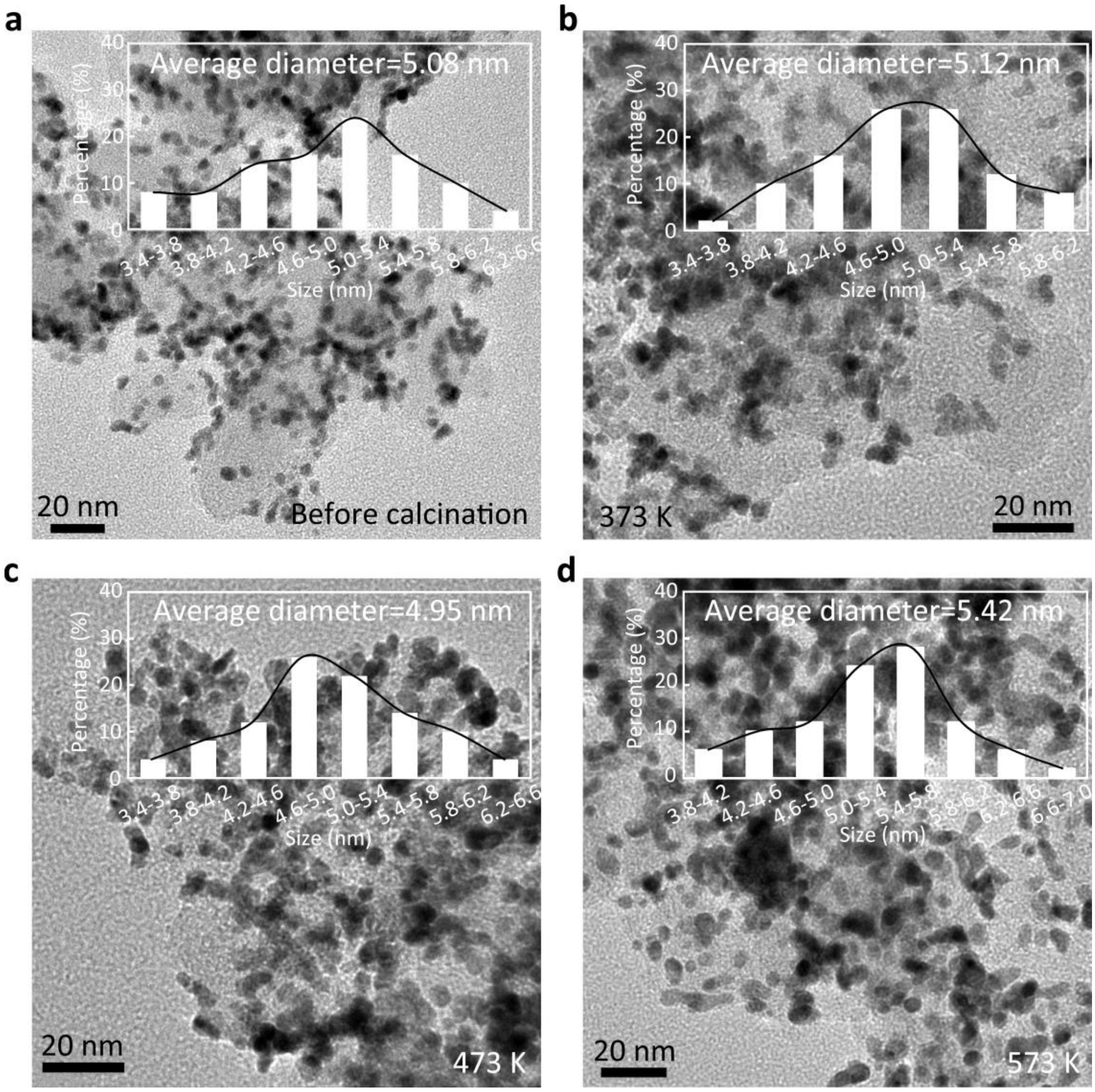

Supplementary Fig. 11. TEM images of Pt/CB a) before calcination, and after calcination for 1 hour at b) 373 K, c) 473 K, and d) 573 K, inset image: the corresponding size distribution histogram of Pt nanoclusters on XC-72 carbon black.

Supplementary Fig. 12. TEM images of Pt NCs

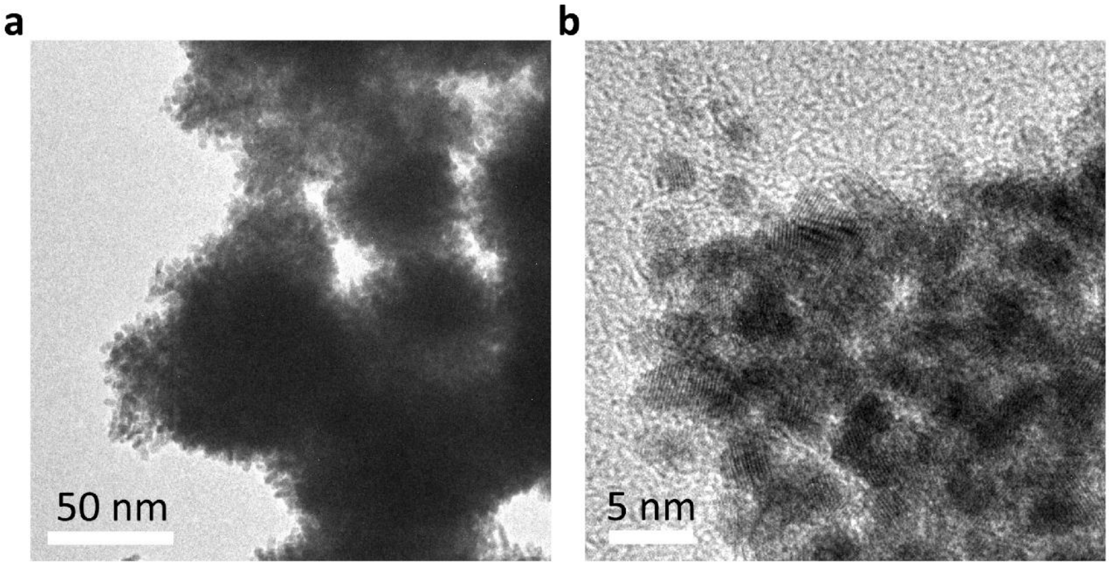

Supplementary Fig. 12. a) TEM image of Pt NCs, b) High-resolution TEM image of Pt NCs.

Supplementary Fig. 13. STEM image and HAADF image of PtC_{60}

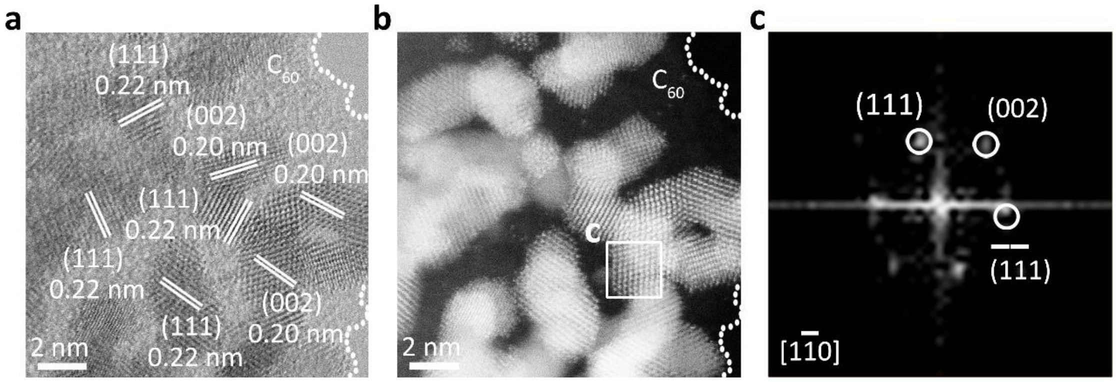

Supplementary Fig. 13. a) STEM image of PtC_{60} and b) the corresponding HADDF of (a), c) FFT pattern of the selected area.

Supplementary Fig. 14. XPS survey spectra of PtC_{60}, Pt NCs, and C_{60} bulk crystal

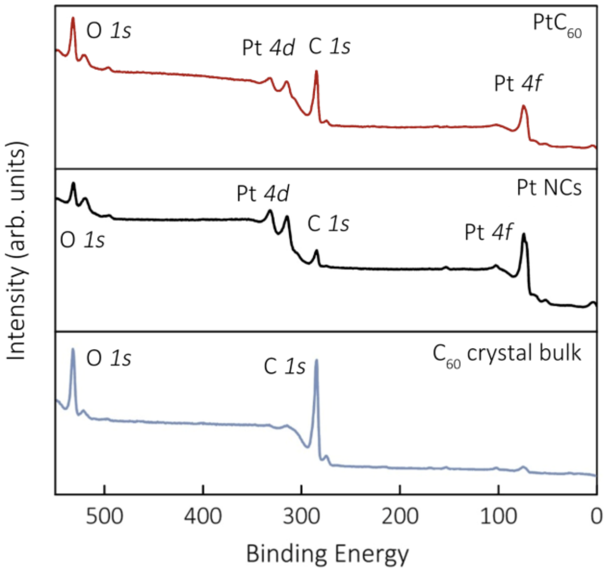

Supplementary Fig. 14. XPS survey spectra of PtC_{60}, Pt NCs, and C_{60} bulk crystal.

Supplementary Fig. 15. XPS O *1s* spectra of PtC_{60}, Pt NCs and C_{60} precursor powder

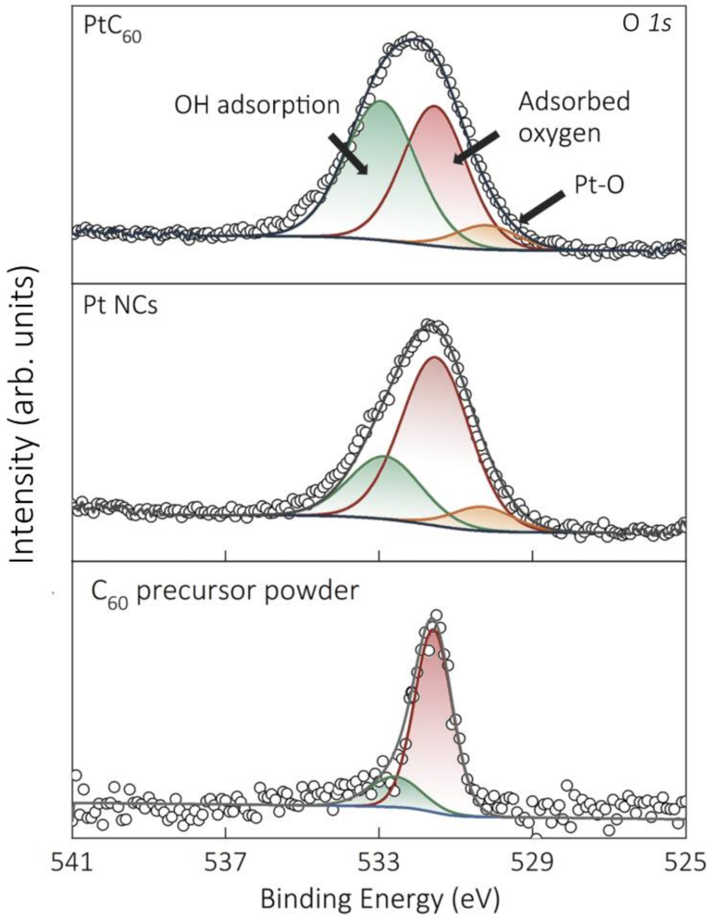

Supplementary Fig. 15. O *1s* of PtC_{60}, Pt NCs and C_{60} precursor powder.

Supplementary Fig. 16. XPS of Pt *4f* of Pt/CB and Pt/C

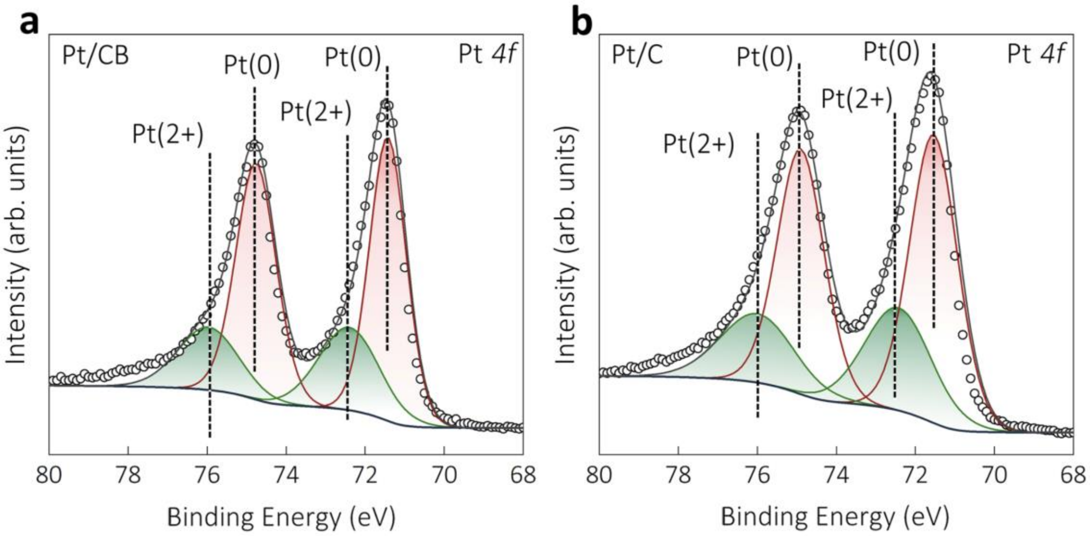

Supplementary Fig. 16. High-resolution XPS of a) Pt *4f* of Pt/CB and b) Pt *4f* of Pt/C.

Supplementary Fig. 17. HER activity and HUPD of Pt/CB with various catalyst loading

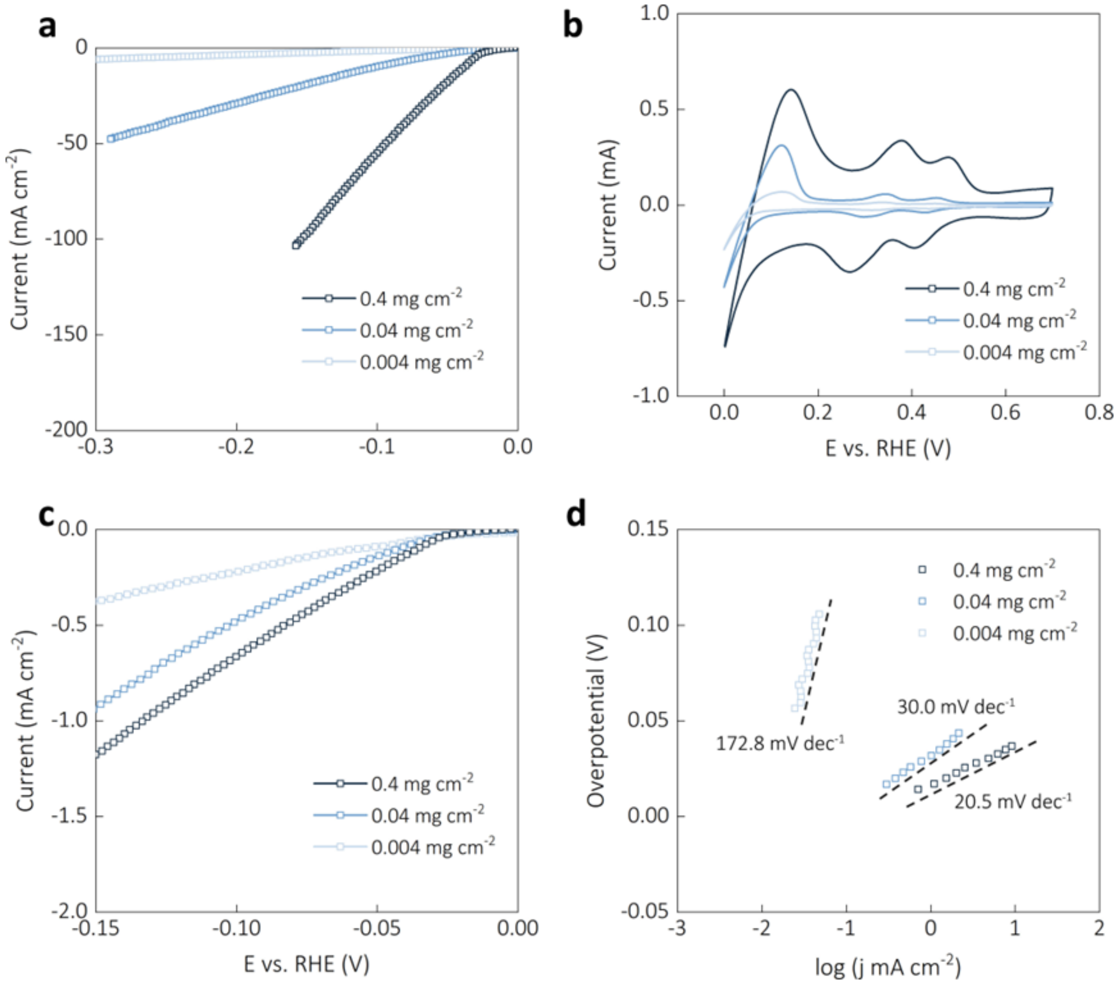

Supplementary Fig. 17. a) LSV curves and b) cyclic voltammetry curves of Pt/CB in N_2-saturated 0.1 M KOH with a scan rate of 50 mV s^{-1}. c) LSV curves of Pt/CB normalized by ECSA with different catalyst loadings. d) corresponding Tafel plots for data in a).

LSV curves of Pt/CB normalized by geometry area demonstrate a similar HER performance as commercial Pt/C (Supplementary Fig. 15a), which is lower than that of the PtC_{60} (Supplementary Fig. 26). To investigate the intrinsic activity of Pt/CB, the ECSA was estimated via measuring the under potential deposited H (Supplementary Fig. 15b, the ECSA data can be viewed in Supplementary Table 3). The LSV curves of

Pt/CB normalized by ECSA (Supplementary Fig. 15c) display a much lower current density than PtC_{60}, which further confirms the improved intrinsic activity of PtC60 towards HER (Supplementary Fig. 31). Similarly to Pt/C and Pt NCs, Tafel slope of Pt/CB increases significantly with decreasing the catalyst loading, from 20.5 to 172.8 mV dec^{-1}, such a high Tafel slope indicates that the HER is limited by Volmer/water dissociation step at low catalyst loading. Overall, based on the electrochemical performance and XPS analysis (Supplementary Fig. 14), it is reasonable to believe that Pt/CB behave similarly with the unsupported Pt NCs.

Supplementary Fig. 18. Schematic illustration of XPS/UPS measurement

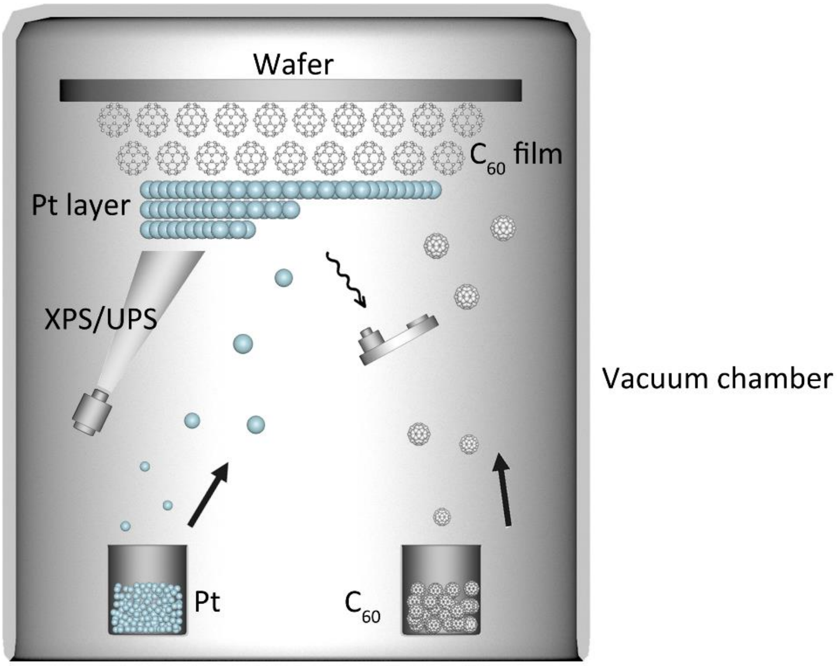

Supplementary Fig. 18. Schematic illustration of the home-built XPS/UPS.

Supplementary Fig. 19. EXAFS spectra and fitting data for PtC_{60} and Pt NCs

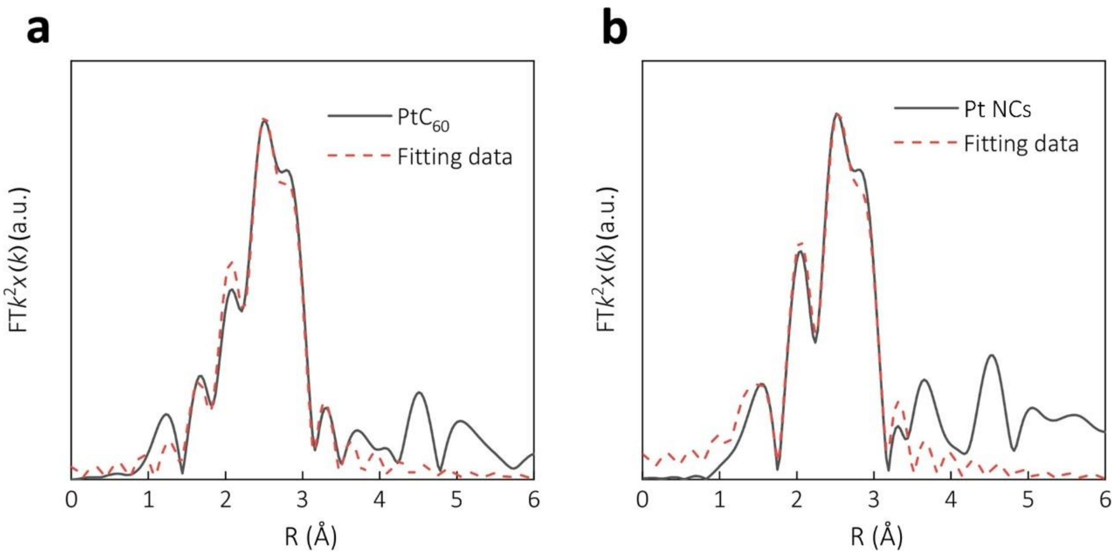

Supplementary Fig. 19. a, b) EXAFS spectra and fitting for a) PtC_{60} and b) Pt NCs at L_3-edge.

Supplementary Fig. 20. Wavelet transform for the k^3-weighted EXAFS spectra of Pt foil and PtO_2

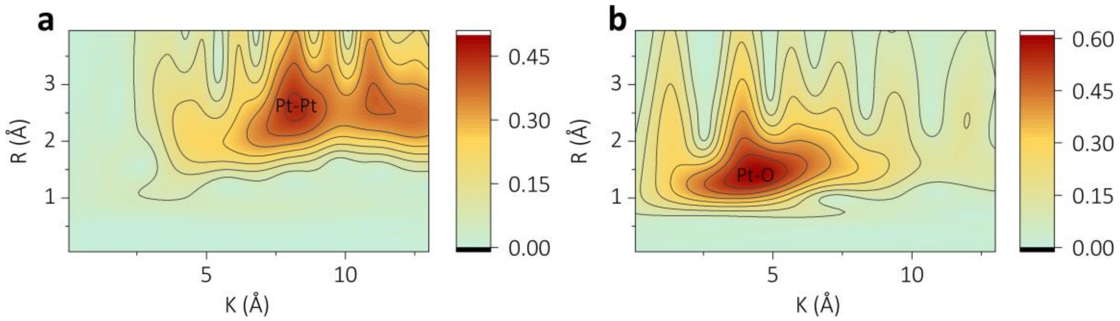

Supplementary Fig. 20. Wavelet transform for the k^3-weighted EXAFS spectra for a) Pt foil, and b) PtO_2.

Supplementary Fig. 21. HER activity of PtC_{60} with various Pt loading in 1.0 M KOH

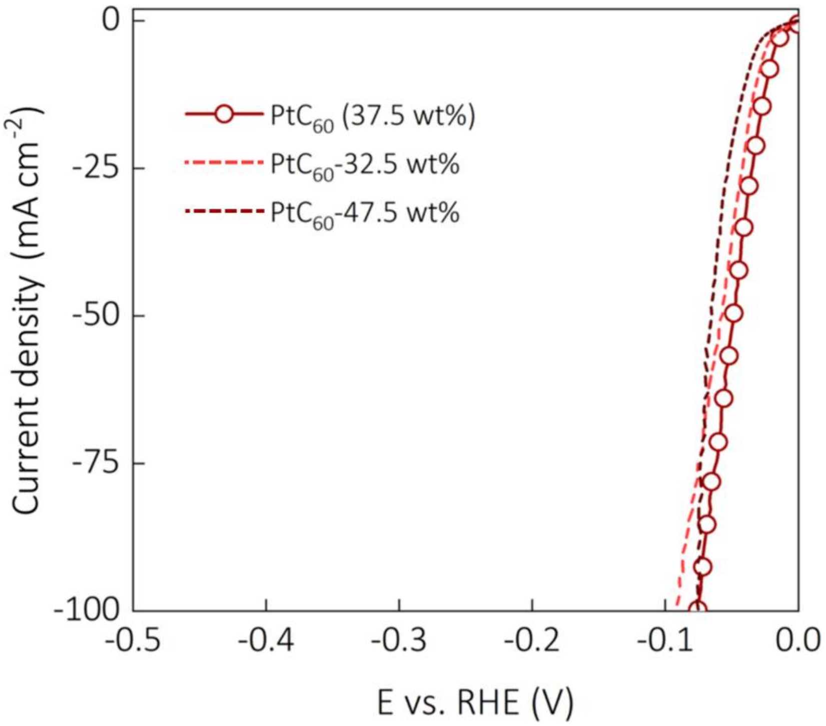

Supplementary Fig. 21. LSV curves of PtC_{60} with various Pt contents in 1.0 M KOH.

Supplementary Fig. 22. Stability performance of PtC_{60} and reference samples

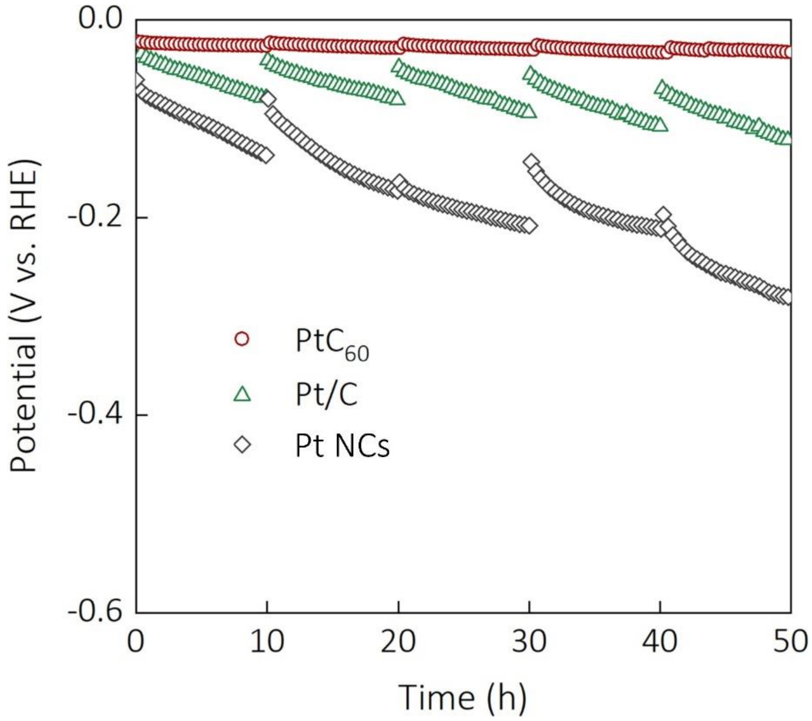

Supplementary Fig. 22. Chronopotentiometry of PtC_{60}, Pt/C, and Pt NCs catalysts on carbon paper in 1.0 M KOH at the current density of 10 mA cm^{-2}.

Supplementary Fig. 23. TEM of PtC_{60} and size distribution of Pt nanoclusters post-test

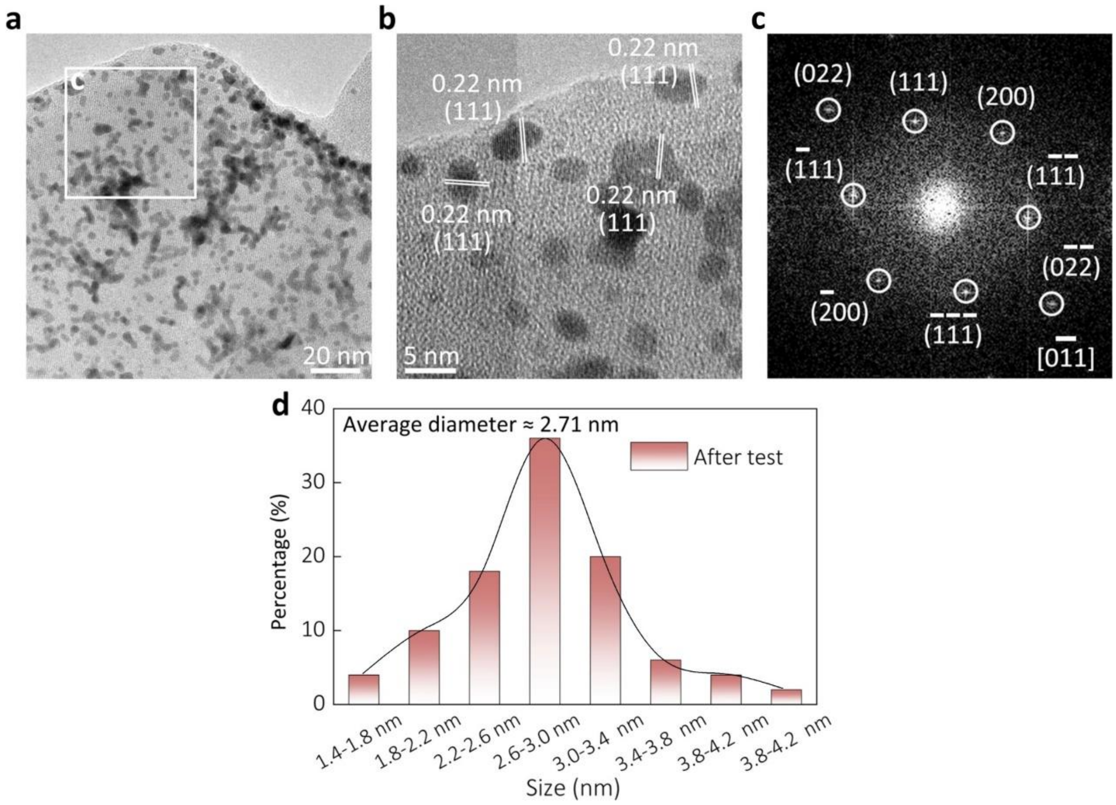

Supplementary Fig. 23. a,b) TEM images of PtC_{60} after 50 cycles of CV test, c) the FFT pattern derived from the selected area in a), d) Size distribution of Pt clusters on C_{60} after 50 cycles of CV test.

Supplementary Fig. 24. Hydrophilicity of PtC_{60}, C_{60} bulk crystal and Pt NCs

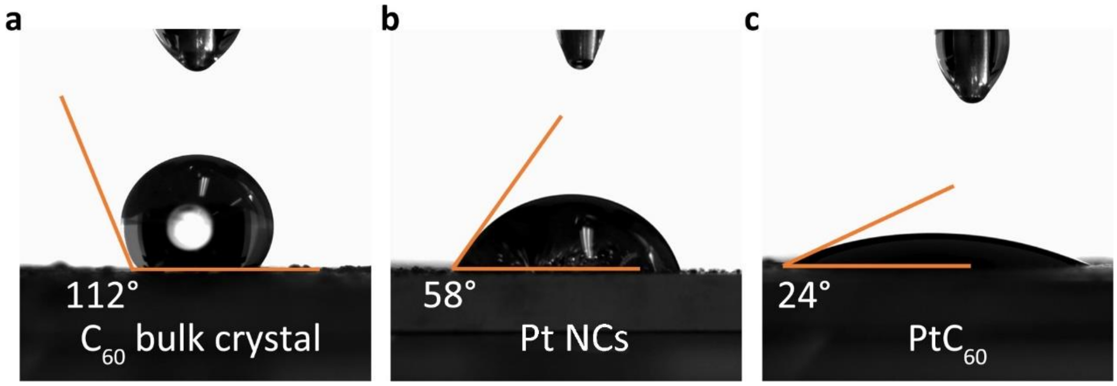

Supplementary Fig. 24. Contact angle measurements of a) C_{60} bulk crystal, b) Pt NCs, and c) PtC_{60}.

Supplementary Fig. 25. Tafel plot of PtC_{60} and reference samples

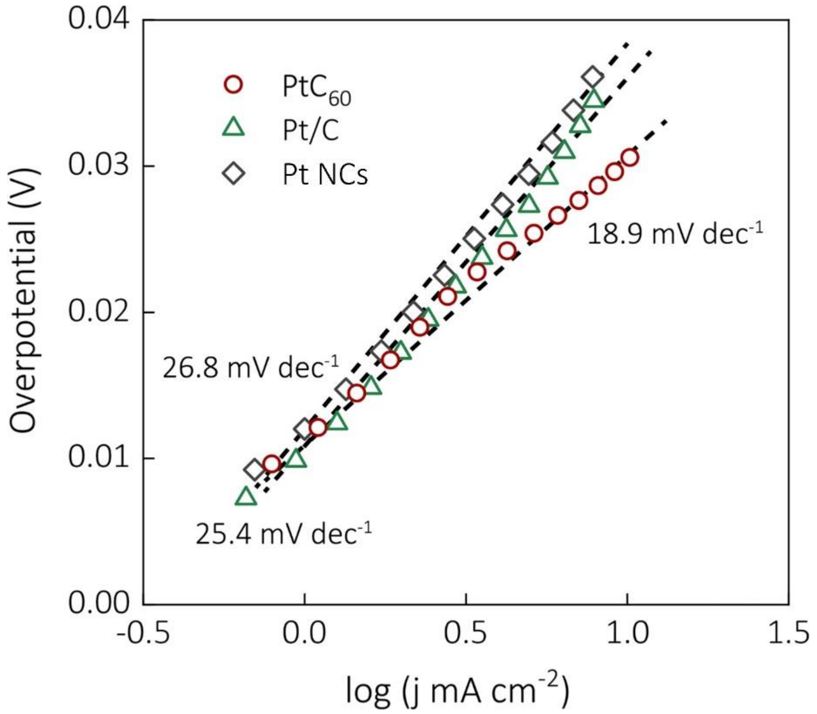

Supplementary Fig. 25. Tafel plot of a) PtC_{60}, b) Pt/C, and c) Pt NCs tested in 1.0 M KOH with a loading of 0.4 mg cm^{-2} normalized by the geometry area of the electrode.

Supplementary Fig. 26. Simulation of HER current-potential curves limited by the inefficient mass transportation

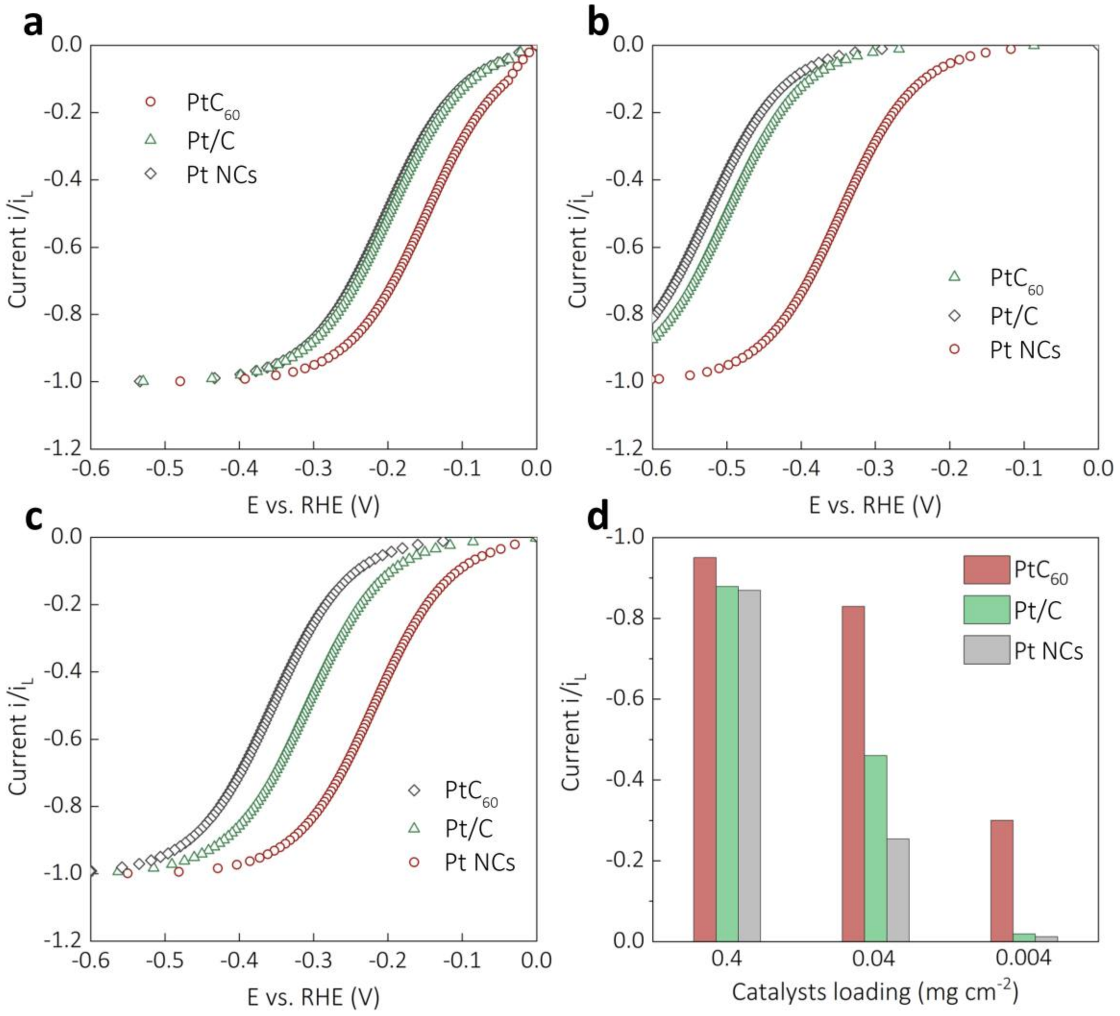

Supplementary Fig. 26. Mass transportation effect: simulated polarization curves of PtC_{60}, Pt/C, and Pt NCs with different loading amounts of a) 0.4 mg cm^{-2}, b)0.04 cm^{-2}, and c) 0.004 mg cm^{-2} catalysts. d) ratio of i to i_L with different catalyst loadings of PtC_{60}, Pt/C, and Pt NCs at -0.3 V vs. RHE.

Supplementary Fig. 27. HER activity of PtC_{60} and reference samples with low-catalyst loadings in 1.0 M KOH normalized by geometry area

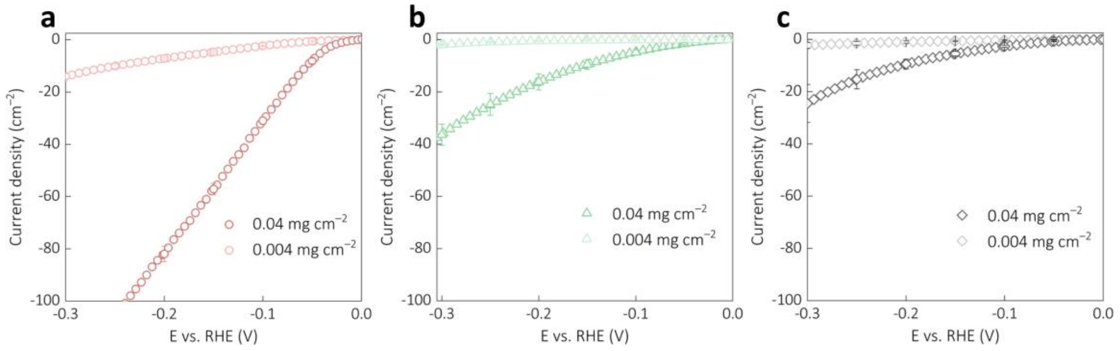

Supplementary Fig. 27. LSV curves of a) PtC_{60}, b) Pt/C, c) Pt NCs normalized by geometry area of electrodes with different catalyst loadings in 1.0 M KOH. Error bars correspond to the standard deviation of three independent measurements.

Supplementary Fig. 28. HER activity of PtC_{60} and reference samples with various catalyst loadings in 1.0 M KOH normalized by Pt mass loading

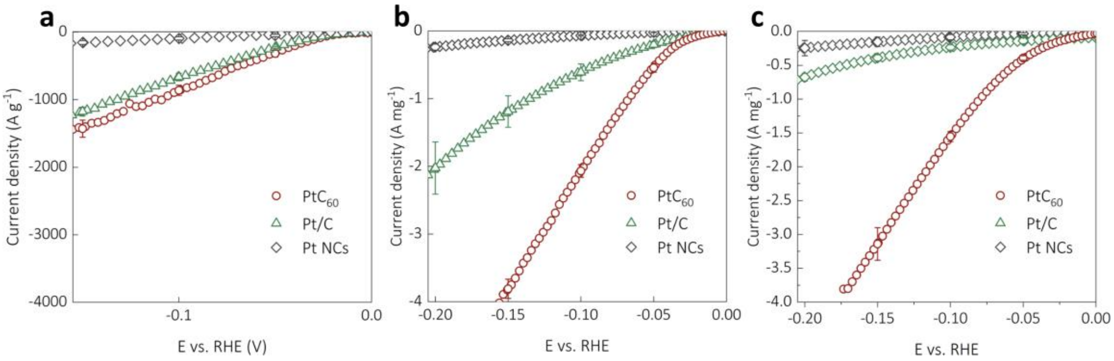

Supplementary Fig. 28. LSV curves normalized by Pt mass loading with different catalyst loadings of a) 0.4 mg cm^{-2}, 0.04 cm^{-2}, and c) 0.004 mg cm^{-2} in 1.0 M KOH. Error bars correspond to the standard deviation of three independent measurements.

Supplementary Fig. 29. HUPD (hydrogen underpotential deposition) of PtC_{60} and reference samples suggested by cyclic voltammetry curves

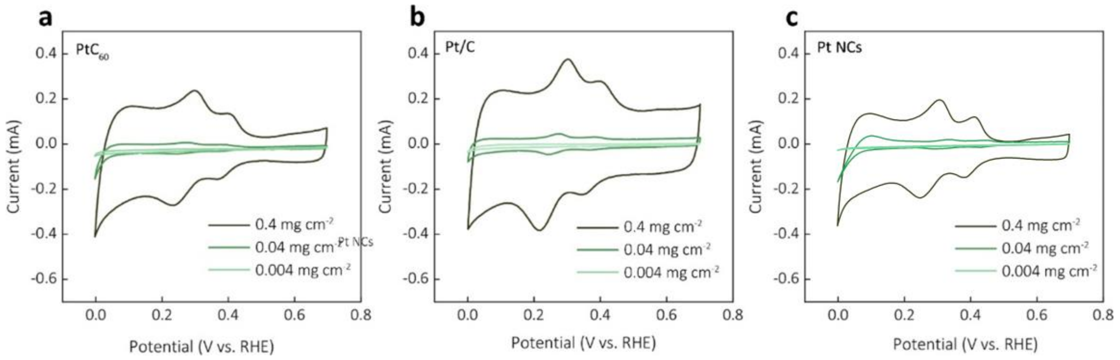

Supplementary Fig. 29. Cyclic voltammetry curves of a) PtC_{60}, b) Pt/C, and c) Pt NCs in N_2-saturated 0.1 M KOH with a scan rate of 50 mV s^{-1}. The calculated electrochemically active surface area (ECSA) can be found in Supplementary Table 2.

Supplementary Fig. 30. HER activity of PtC_{60} and reference samples with various catalyst loadings in 1.0 M KOH normalized by ECSA

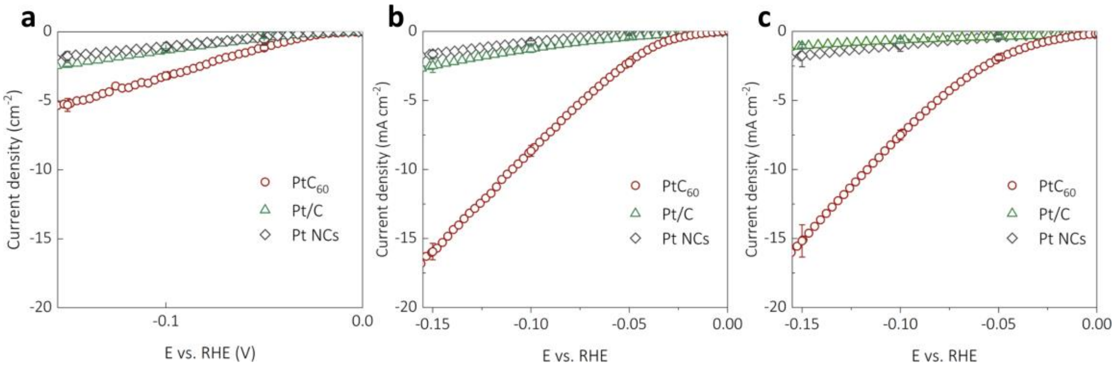

Supplementary Fig. 30. LSV curves normalized by ECSA with different catalyst loadings of a) 0.4 mg cm^{-2}, b) 0.04 cm^{-2}, and c) 0.004 mg cm^{-2} in 1.0 M KOH. Error bars correspond to the standard deviation of three independent measurements.

Supplementary Fig. 31. Tafel plot of PtC_{60} and reference samples with different catalyst loadings normalized by electrode geometry area in 1.0 M KOH

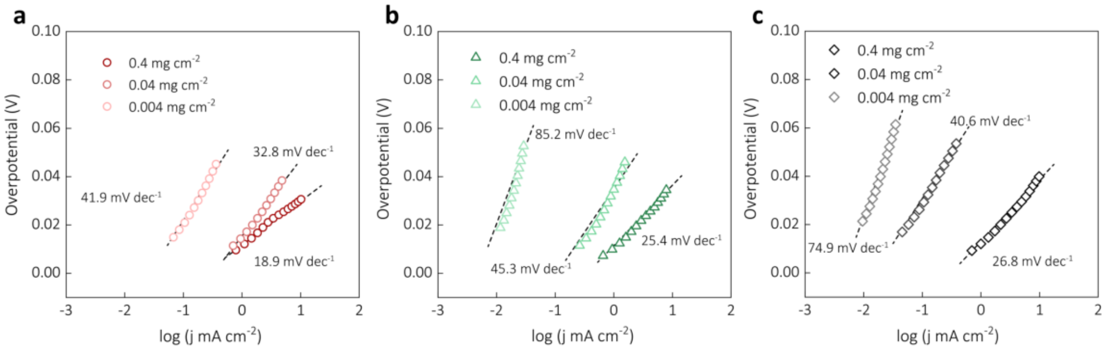

Supplementary Fig. 31. Tafel plot of a) PtC_{60}, b) Pt/C, and c) Pt NCs normalized by electrode geometry area with different catalyst loading in 1.0 M KOH.

Supplementary Fig. 32. HER activity of PtC_{60} and reference samples with various catalyst loading in 0.5 M H_2SO_4 normalized by geometry area

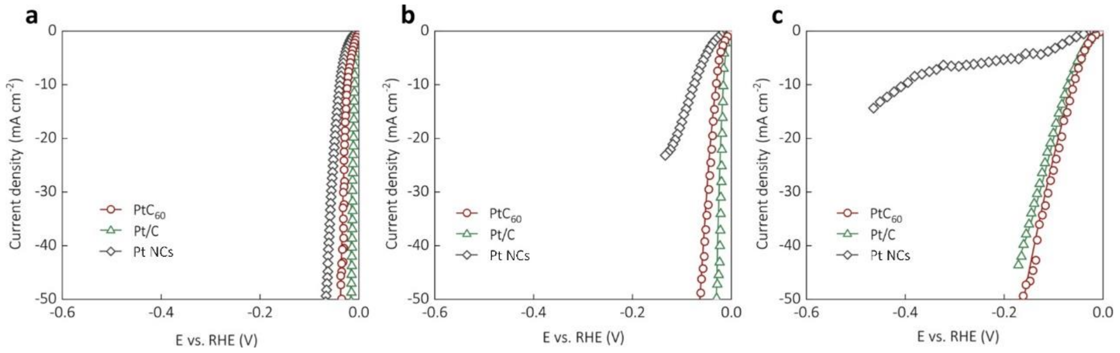

Supplementary Fig. 32. LSV curves normalized by geometry area of electrode with different catalyst loadings of a) 0.4 mg cm^{-2}, b) 0.04 cm^{-2}, and c) 0.004 mg cm^{-2} in 0.5 M H_2SO_4.

Supplementary Fig. 33. Nyquist plots of PtC_{60}, Pt/C, and Pt NCs in 1.0 M KOH at −40 mV vs. RHE.

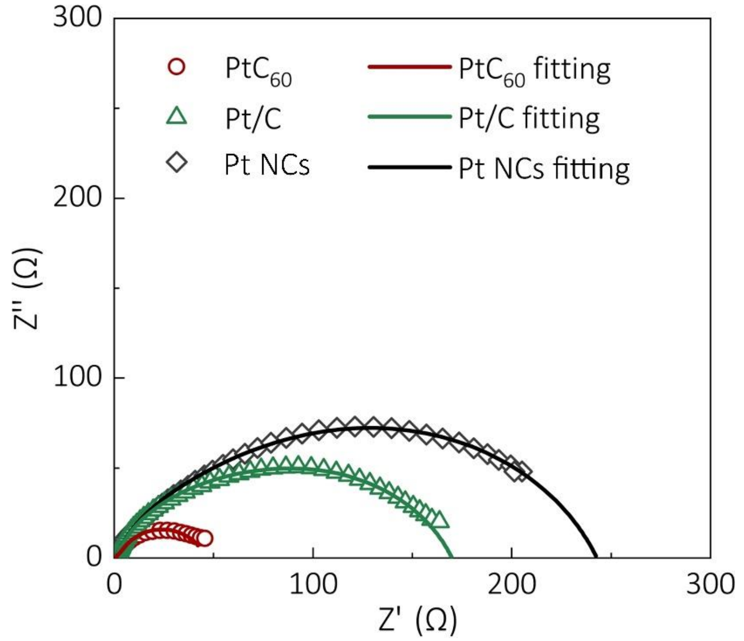

Supplementary Fig. 33. Nyquist plots of PtC_{60}, Pt/C, and Pt NCs in 1.0 M KOH at −40 mV vs. RHE, the dots represent the experimental data collected, while the solid lines correspond to the curve fitting. The fitting results are shown in Supplementary Table 4.

Supplementary Fig. 34. Correlation between MEAM and DFT binding energies.

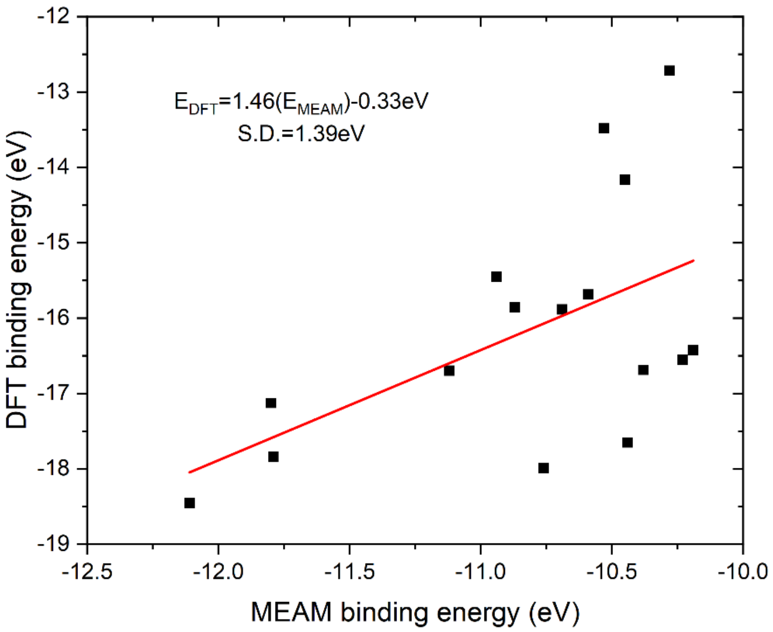

Supplementary Fig. 34. Correlation between MEAM and DFT binding energies for the 16 most stable structures of Pt/C_{60}(011) calculated with MEAM interatomic potentials.

Supplementary Fig. 35. Bader charge on Pt atoms in Pt/C_{60} model

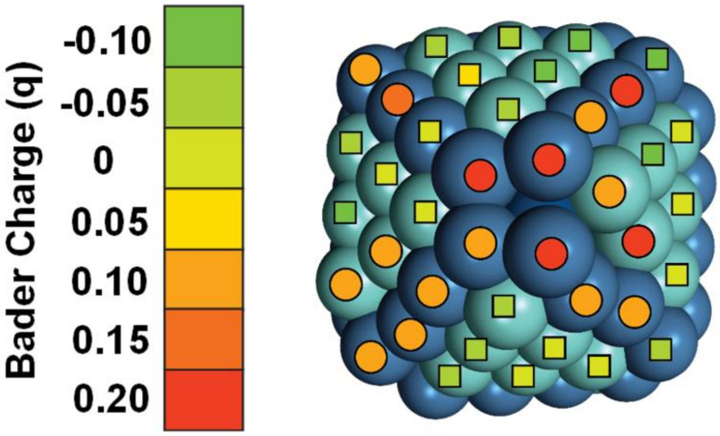

Supplementary Fig. 35. Bader charges on Pt atoms on the side of Pt particle that is in contact with the C_{60}(011) surface. Bader charges on Pt atoms in contact with the C_{60} support are represented by circles, whereas charges on atoms not in contact with the C_{60}(011) surface are represented by squares.

Supplementary Fig. 36. Calculated work functions of Pt NCs

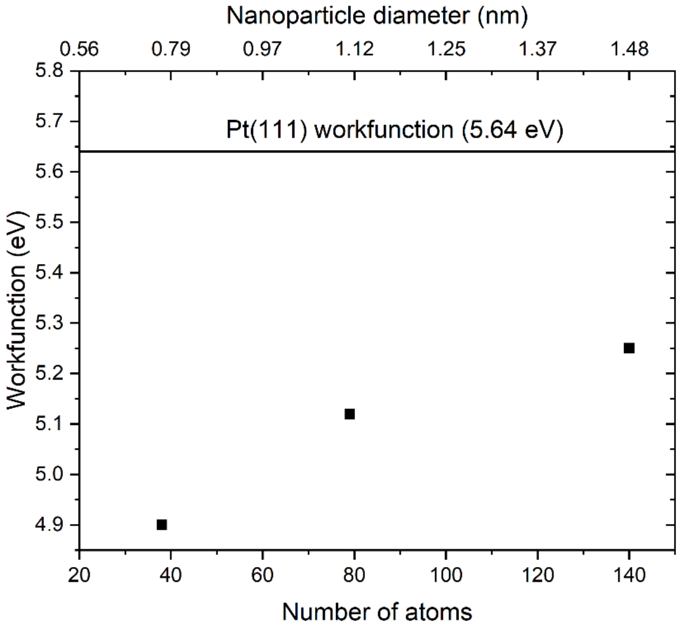

Supplementary Fig. 36. Calculated work functions of Pt nanoparticles as a function of their size.

Supplementary Fig. 37. Density of states (DOS) per atom for the carbon atoms.

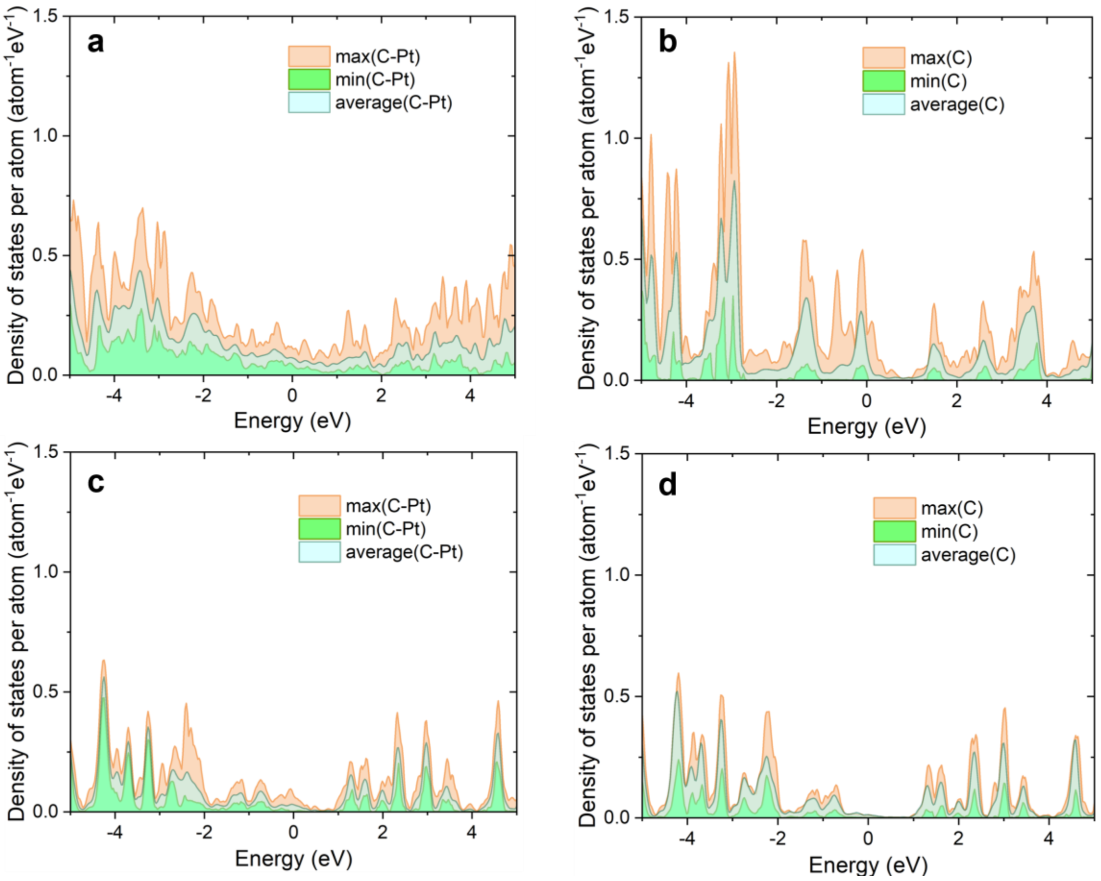

Supplementary. Fig. 37 Maximum, minimum and average value of the density of states (DOS) per atom for a) the carbon atoms bonded to the Pt (C-Pt) on Pt/C_{60}, b) carbon atoms not bonded to Pt on Pt/C_{60}, c) carbon atoms bonded to the (C-Pt) on graphene, d) carbon atoms not bonded to Pt on Pt/graphene.

Supplementary Fig. 38. Pt NC adsorbed on C_{60}(011) and Pt/graphene systems.

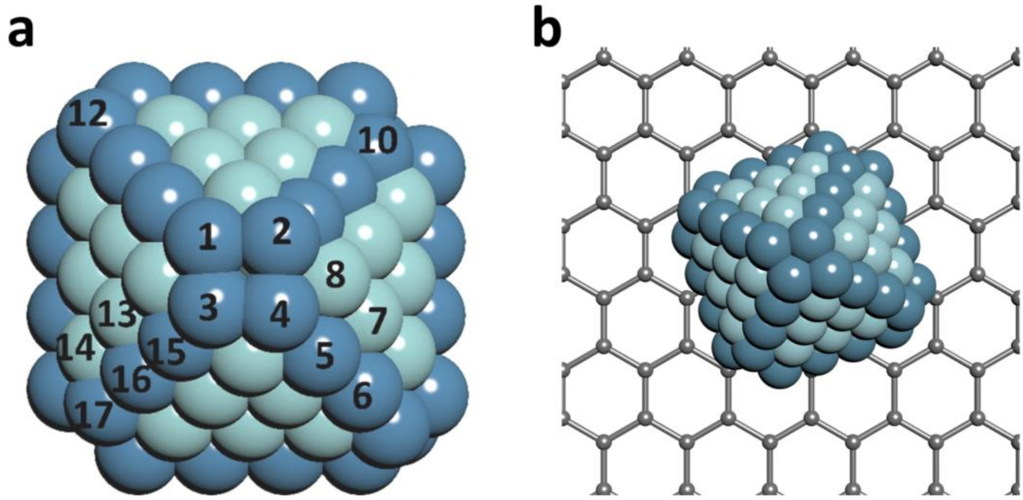

Supplementary Fig. 38. a) The geometry of Pt nanoparticle adsorbed on C_{60}(011) with the numerated Pt atoms forming the Pt/C_{60} interface. The fullerene molecules are not displayed for clarity. b) The geometry of Pt NC adsorbed on graphene surface.

Supplementary Fig. 39. Color Map to illustrate reaction rate constant on different atop Pt sites for Pt/C_{60} (011) for different forward alkaline HER reaction steps.

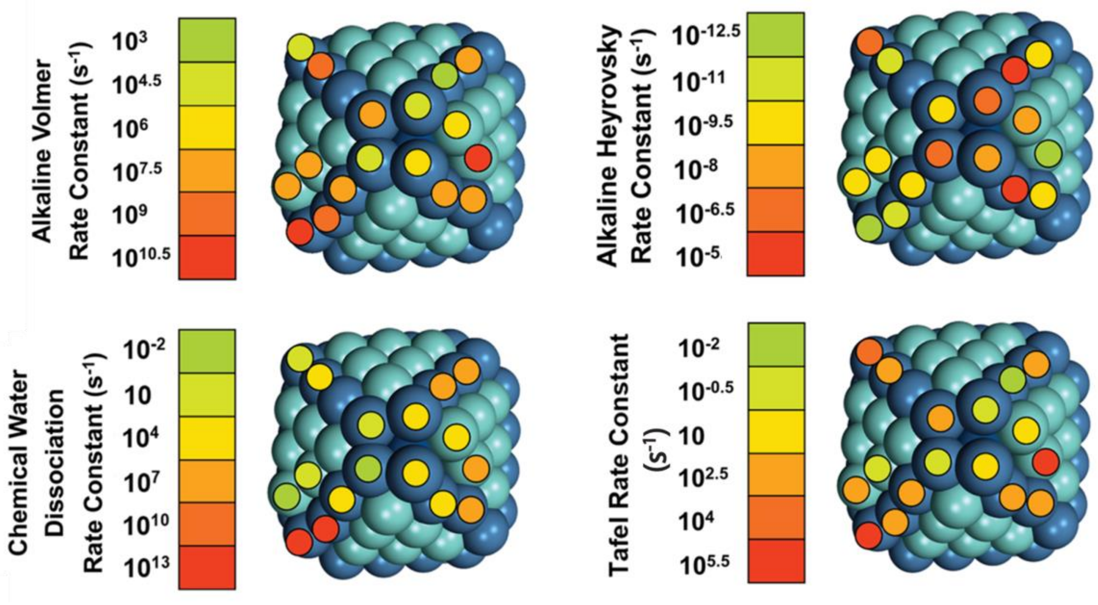

Supplementary Fig. 39. Reaction rate constants for various alkaline HER forward reaction steps on Pt sites on the interface of Pt/C_{60} (011). The fullerene molecules are not displayed for clarity.

Supplementary Fig. 40. Gibbs free energy profile for H diffusion

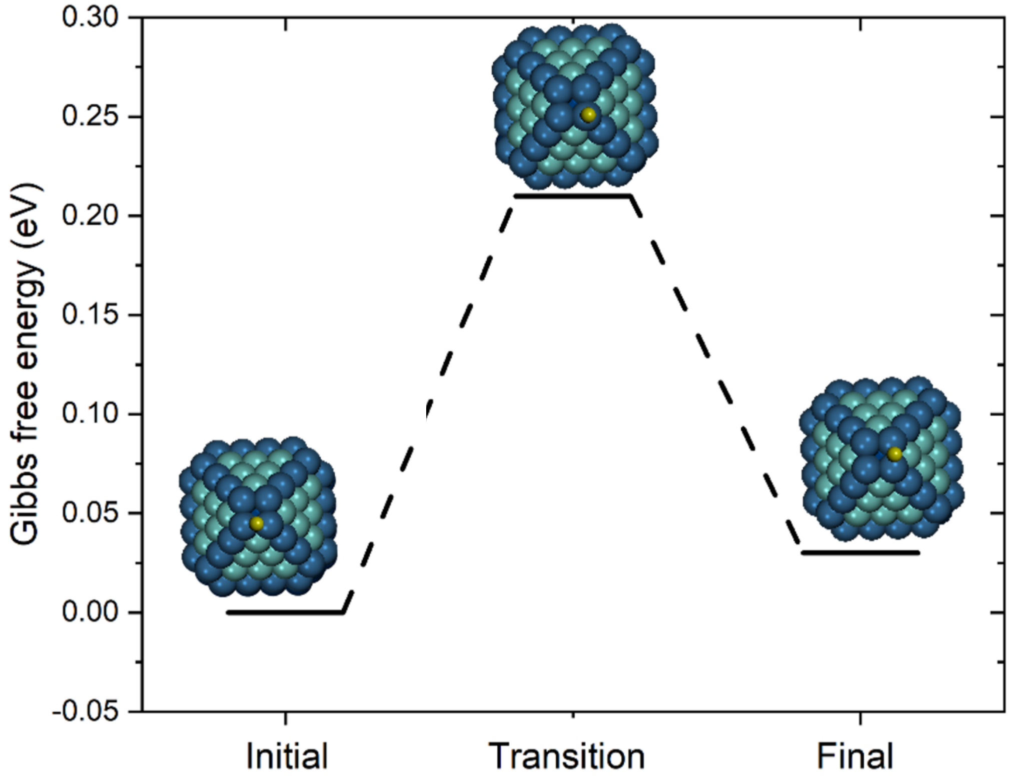

Supplementary Fig. 40. Gibbs free energy profile for H diffusion between 2 bridge positions on unsupported Pt NC.

Supplementary Fig. 41. Simulated LSV for 17 Pt surface interfacial sites on Pt/C_{60} (011), 16 Pt surface interfacial sites on Pt/graphene, and unsupported Pt NCs

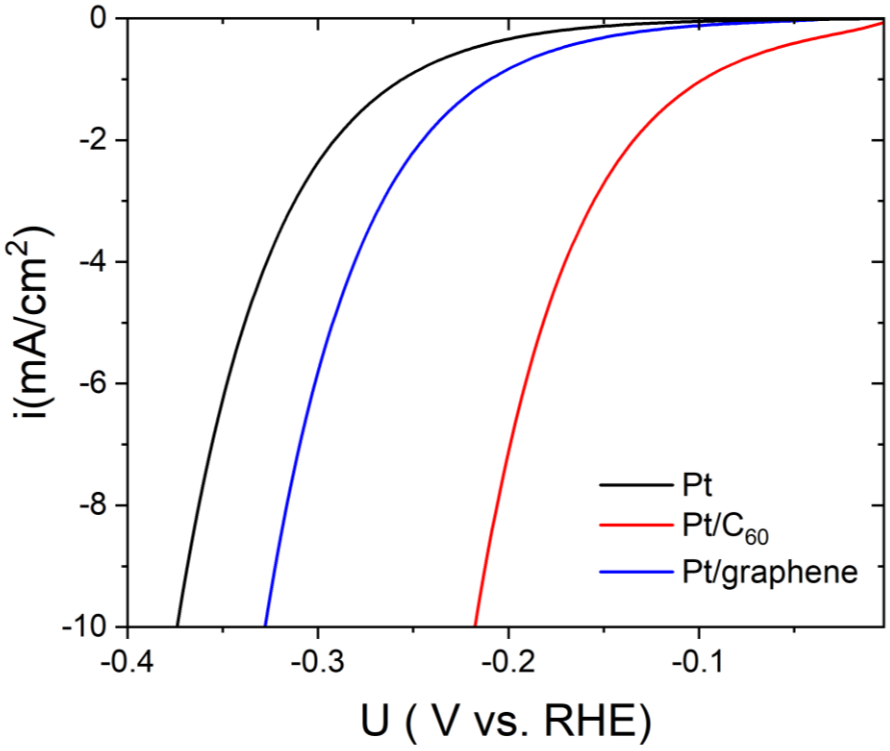

Supplementary Fig. 41. Simulated LSV for the 17 interfacial Pt-sites at the Pt/C_{60} (011) interface, 16 interfacial Pt-sites at the Pt/graphene interface, and all Pt-sites on unsupported Pt NCs normalized per number of Pt atoms.

Supplementary Fig. 42. Photograph of the AEM electrolyser

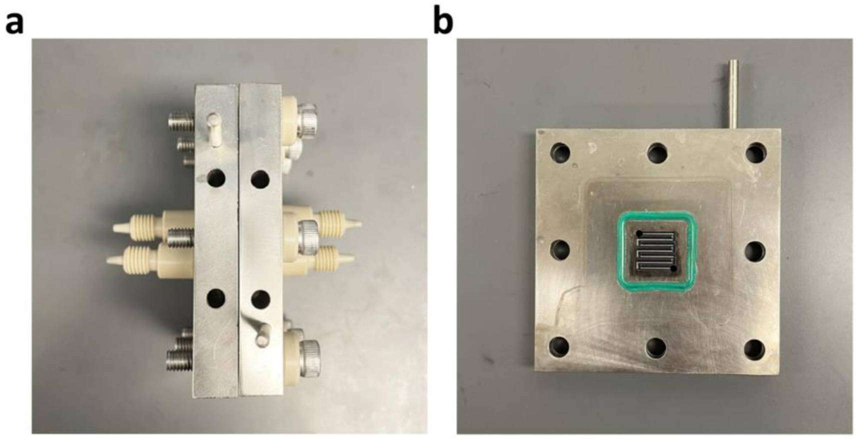

Supplementary Fig. 42. a) Photo of the assembled AEM electrolyser, b) photo of the disassembled AEM electrolyser (cathode).

Supplementary Fig. 43. **Fitting procedure of UPS spectra during Pt deposition on C_{60} film**

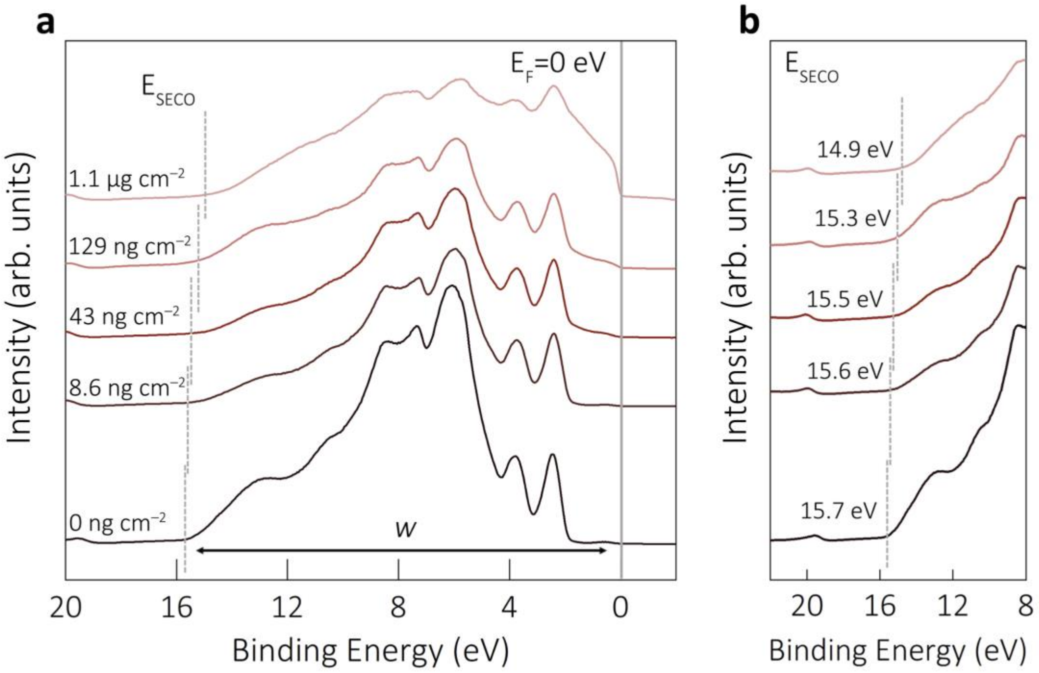

Supplementary Fig. 43 a) UPS spectra and b) high-resolution UPS spectra during Pt deposition on C_{60} film

Supplementary Notes

Supplementary Note 1. Effect of hydrophilicity on catalytic stability

We observed that PtC_{60} exhibits improved hydrophilicity compared with the Pt NCs. Specifically, the contact angles of the C_{60} bulk crystal, Pt NCs and PtC_{60} were determined to be 112°, 58° and 24°, respectively, as shown in Supplementary Fig. 24a-b. As a result, we believe the enhanced hydrophilicity of PtC_{60}, which inhibits the formation of large H_2 bubbles, can prevent the physical structural damage of the catalyst layer caused by bubble formation,[4–6] and further contribute to its long-term stability for HER.

Supplementary Note 2. Mass diffusion effect

Sluggish mass transport on electrode surface is an important issue to consider during investigations of the intrinsic activity of Pt-based catalysts toward HER. Even under RDE experiment, the H_2 evolution is so fast and the measured current can be easily limited by mass transport of H_2 away from the electrode.[7,8] The model of Hansen et al. displays that the intrinsic activity of Pt under acidic medium on RDE system has no effect on the catalytic performance. The frequently reported Tafel slope of 30 mV/dec for HER is just the apparent Tafel slope of the diffusion controlled overpotential. Thus, mass-transport limitations prevent a genuine comparison of intrinsic activities. In this work, we adapted the strategy of decreasing the catalyst loading to mitigate the influence of mass-transport effects in evaluation of catalysts.

Due to the fast evolution of H_2 molecules under the high current density (*e.g.*, i > 2 mA cm^{-2}),[7,9] the H_2 gas oversaturation could cause HER kinetics primarily limited by the inefficient mass transportation, especially with under slow scan rate. This process is called Nernstian reaction, and limited current follow the Levich equation as in Eq. 1:[7]

$$i_l = 0.62n\ F\ A\ aD^{2/3}\upsilon^{-1/6}\ c_0\ \omega^{1/2} \qquad [1]$$

The electrode polarization curves follow the Nernstian reaction in Eq. 2:[8]

$$\frac{i}{i_0} = \left(1 - \frac{i}{i_l}\right) e^{-\alpha f\eta} - \left(1 - \frac{i}{i_l}\right) e^{(1-\alpha)f\eta} \qquad [2]$$

where i_l is the mass transport limiting current, D is the diffusivity of hydrogen in the electrolyte, n is the electron transfer number in HER (n = 2), A is the area of the electrode (0.1256 cm^{-2}), a is the diffusion coefficients, hypothesize to equal a =0.01, D ($3.03*10^5$ cm2 sec^{-1}) is the diffusion coefficient of H_2, υ (0.998×10^{-2} cm $^{2}\cdot s^{-1}$) is the kinematic viscosity of the aqueous electrolyte, c_0 (12.13 ml/L ≈ 0.5415 mM) is the solubility of H_2 in the aqueous electrolyte under room temperature, ω (1600 rpm) is the electrode rotation rate, α (0.5) is transfer coefficient. f=F/RT, F is Faraday constant, R is universal gas constant, and T (298 K) is reaction temperature.

Based on the above model, the i_l is calculated to be 0.0316 A. The i_0 is estimated from polarization curves as indicated in Supplementary Table 2. As shown in Supplementary Fig. 26a, the facile electrode kinetic of PtC_{60} is limited by the diffusion of produced H_2 when a typical catalyst loading was used. Due to the Nernstian limitation, the intrinsic kinetics plays a minor role under these catalyst loadings with large exchange currents for all the samples, in which i/i_l at –0.3 V vs. RHE is –0.95, –0.88, and –0.87 for PtC_{60}, Pt/C, and Pt NCs (Supplementary Fig. 26d), respectively. As a result, all polarization curves stack together showing a small discrepancy of the HER activity. However, the interval of the corresponding polarization curves increases along with the decrease in catalyst loading (Supplementary Fig.26b-c). At the loading of 0.004 mg cm^{-2}, the i/i_l of PtC_{60} at –0.3 V vs. RHE is 0.3, which is ~15 times of Pt/C, and ~24 times of Pt NCs (Supplementary Fig. 26d). In this case, the slower overall HER production rate at low catalyst loading mitigates the influence of mass-transfer effect of H_2, so that profound differences in HER kinetics can be observed among these Pt catalysts with different intrinsic activities. Thus, it is more appropriate to investigate the intrinsic activity of PtC_{60} and the reference samples using low-catalyst loadings.

Supplementary Note 3. Design and optimization of the calculated catalyst model

The model employed in the theoretical calculations was designed based on the

experimental observations. In our models, unsupported platinum NCs and Pt NCs on C_{60} support are represented by a truncated octahedron with an fcc structure composed of 140 metal atoms with a size of ~1.4 nm, which resembles experimentally observed clusters (Supplementary Fig. 4). In turn, the substrate is represented by the $\sqrt{8}x\sqrt{8}$ supercell of (011) surface of a C_{60} nanosheet with the fcc structure, constructed based on the experimental lattice parameter of a = 14.14 Å for fullerene crystals.[10] Such supercell had 24.5 × 24.5 Å lateral dimensions and 13 Å slab thickness (2 C_{60} layers) resulting in >9 Å separation between periodically repeated Pt particles. In turn, the vacuum in the direction perpendicular to the fullerene (011) surface amounted to 12 Å. The realistic model of Pt/C_{60}(011) catalysts with high thermodynamic stability was created by identifying the lowest energy structure among a multitude of tentative structures of Pt/C_{60}(001) interfaces. We generated such structures via a systematic l×n×m grid scan, where we displaced the particle by $r_i = i * \frac{a}{l}, i \in [0, l)$ along the funnel of C_{60}(011) surface and rotated it by $\phi_j = j * \frac{2\pi}{n}, j \in [0, n)$ and $\theta_k = k * \frac{2\pi}{m}, k \in [0, m)$ degrees around the axes passing through its centre of mass and perpendicular or parallel to the surface. In the present study, we examined 5×5×5, 7×7×7, 9×9×9, and 11×11×11 grids. Increasing the grid size decreased the energy of the lowest energy obtained isomer by less than 0.04 eV. These initial geometries were relaxed by employing the Modified Embedded Atom Method (MEAM) potential developed by Byeong-Joo Lee et al,[11] which is implemented in the Large-scale Atomic/Molecular Massively Parallel Simulator (LAMMPS) package.[12] The calculations were performed keeping the surface fixed to keep the lattice parameters and avoid the distortion of the slab. The threshold parameters for geometry optimization in LAMMPS calculations were $1 \times 10^{-8} eV$ for the energy and $1 \times 10^{-8} eV/Å$ for the forces. The graphene surface has been modelled by a 10×10 graphene supercell, constructed based on experimental lattice parameter of a = 2.46 Å. Such a supercell has 24.6 Å × 24.6 Å lateral dimensions, resulting in > 9 Å separation between periodically repeated Pt particles. Subsequently, the vacuum perpendicular to the graphene surface

amounted to 12 Å . In turn, the structure of Pt nanoparticles on graphene surface was chosen to be similar to the most energetically stable structure of Pd nanoparticles supported on graphene obtained in previous studies.

Supplementary Note 4. DFT optimization of Pt/C_{60} structures

16 most stable systems calculated with MEAM were relaxed by means of electronic structure calculations using Vienna Ab-initio Simulation Package (VASP),[13,14] with the generalized gradient approximation (GGA) PBE functional.[15] The VASP calculations were performed employing only the gamma point in the reciprocal space. The interactions between core and valence electrons were described with the projector augmented wave (PAW) approach using a plane-wave basis set with the cut-off energy of 400 eV. The dispersion interaction between the atoms is also considered by means of the D3 method developed by Grimme.[16] The Methfessel-Paxton smearing method with the smearing width of 0.1 eV was employed to set the partial occupancies for each orbital. The threshold for the SCF calculation is $1 \times 10^{-5} eV$ for the changes in the energy, while the geometric relaxations of all the atoms were terminated once the norms of the forces acting on all atoms were smaller than $2 \times 10^{-2} eV/Å$.

Supplementary Fig. 34 shows the correlation between the binding energies of Pt onto C_{60} surface calculated with DFT and MEAM, where the binding energies are:

$$E_b(Pt_{140}/C_{60}(011)) = E(Pt_{140}/C_{60}(011)) - E(Pt_{140}) - E\big(C_{60}(011)\big) \quad [3]$$

Here $E(Pt_{140}/C_{60}(011))$ is the total DFT energy of the Pt NCs adsorbed on the slab and $E(Pt_{140})$, $E\big(C_{60}(011)\big)$ are the total DFT energies of the Pt NCs and pristine slab, respectively. With this definition, the negative adsorption energy values correspond to the exothermic binding. The most energetically stable structure of Pt/C_{60} interface was calculated to be the same according to both VASP and LAMPPS calculations. Since we performed VASP calculations for all MEAM structures within 2 eV from the structure with the lowest energy, we expect the obtained model of Pt/C_{60} to be close to the true constrained global minimum for Pt nanocrystallites supported by C_{60}. The identified low energy structure will be used in further studies of the binding energies and reactivity of Pt/C_{60} system.

Supplementary Note 5. Analysis of the electron transfer through the Pt/C_{60}(011) interface

To qualitatively characterize the electron density at the Pt|C_{60}(011) interface, we calculated the polarization of the electron density $\Delta\rho$ via charge density difference method. Namely, Supplementary Fig. 4b in the main text displays the isosurfaces of the difference between the electron densities of the lowest-energy optimized Pt/C_{60}(011) model and the electron densities of separated Pt particle and C_{60}(011) calculated in a single-point fashion using atomic positions from the optimized Pt/C_{60}(011) complex. To quantify the perturbation in the electron density, we integrated it over (x,y) planes parallel to the C_{60} surface (Supplementary Fig. 4b in the main text):[9]

$$\Delta\lambda(z) = \iint_{cell} \rho_{diff} dxdy \qquad [4]$$

In turn, the dipole moment of Pt/C_{60} was calculated by integrating the function $z\Delta\lambda(z)$ and multiplying it by the negative electron charge q_e across the cell:

$$\Delta\mu_N = q_e \int z\Delta\lambda(z)dz \qquad [5]$$

The Pt atoms bonded to the C atoms in C_{60}(011) have a positive charge indicating, that it is an electron donor. The Pt atoms not bonded to C atoms in C_{60}(011) mostly donate electrons to the support, although some of them also obtain negative charges (Supplementary Fig. 35).

Supplementary Note 6. The work function of the considered Pt/C_{60} model

The calculated work function for the Pt NCs is 5.25 eV, which is smaller than 5.64 and 5.62 eV obtained for the Pt(111) and Pt(100) surfaces, respectively, but larger than the work functions of smaller NCs, 4.90 eV for Pt_{38} and 5.12 eV for Pt_{79} (as can be seen in Supplementary Fig. 36). In turn, the calculated work function for the C_{60}(011) surface is 5.50 eV, which is the same as observed in our experimental results. These results suggest a significant driving force for the electron transfer from the Pt NCs to the fullerene surface once the interface is formed. Nevertheless, a more detailed analysis of the electron density at the Pt|C_{60}(011) interface described earlier in the manuscript reveals that the dipole moment on the interface points towards the fullerene surface, which increases the work function of the entire system to 6.00 eV. Similarly, high work function of Pt/C_{60} nanocomposite was also observed in our experiments.

The work function for the Pt NCs in contact with the graphene surface is 5.15 eV and the Bader charge analysis for the 12 carbon atoms bonded to Pt atoms shows a charge of 0.15 e^- from the Pt nanoparticle to the graphene surface. Much lower value of the work function and Bader charges in Pt/graphene system corroborates much stronger electronic interactions of Pt particles with C_{60} support than graphene support.

Supplementary Note 7. Molecular adsorption

The alkaline HER mechanism involves H_2O, OH, and H species, whose binding energies to the catalyst govern the HER reaction rate. The adsorption of H_2O is preferred on atop positions while adsorption of H and OH is possible on both atop and bridge adsorption sites, both of which are calculated for the sake of comprehensiveness. The adsorption of H_2O on Pt is strengthened in the presence of fullerene support as some Pt atoms on Pt/C_{60} interface exhibit more exothermic H_2O binding. The Gibbs free adsorption energies of these 3 species are also calculated for the Pt sites on Pt/graphene interface and unsupported Pt NCs, and compared with those of the Pt-sites on Pt/C_{60}.

The Gibbs free energies of adsorbed species are calculated considering the vibrational modes of the species adsorbed by utilizing the harmonic oscillator model. The Gibbs free energy of the gas phase species is calculated by the ideal gas model which considers the vibrational frequencies, the symmetry number, the rotation of the system, and the temperature and pressure of the reaction conditions. The thermodynamic analysis is performed for room temperature (298.15 K) while the pressure is set to 1 bar for H_2 gas and 0.03534 bar for H_2O, which corresponds to the saturated vapor pressure at 298.15 K. Note that the Gibbs energies of water molecules in the vapor phase and liquid phase are equal at this vapor pressure. The Gibbs free energies of adsorption for the different species are calculated using the Eq. 6 to 10 shown below:

$$G_{eff}(H) = 0.5 \times G(H_2) \quad [6]$$

$$G_{eff}(OH) = G(H_2O) - 0.5 \times G(H_2) \quad [7]$$

H_2O adsorption :

$$G_{ads}(H_2O/Pt_x) = G(H_2O/Pt_x) - G(Pt_x) - G(H_2O) \quad [8]$$

H adsorption :

$$G_{ads}(H/Pt_x) = G(H/Pt_x) - G(Pt_x) - \frac{1}{2}G(H_2) \quad [9]$$

OH adsorption :

$$G_{ads}(OH/Pt_x) = G(OH/Pt_x) - G(Pt_x) - G_{eff}(OH) \qquad [10]$$

Where $G(H_2O/Pt_x), G(H/Pt_x),\ G(OH/Pt_x)$ are the total Gibbs free energy of the H_2O, H and OH adsorbed to the catalyst ($Pt_{140}/C_{60}(011)$, Pt/graphene or unsupported Pt), respectively, $G(Pt_x) = E(Pt_x)$ is the total DFT energy of the catalyst model and $G(H_2O), G(H_2)$ are the total Gibbs free energies of a water and hydrogen molecules, respectively. The Gibbs free effective energies of the H and OH adsorbate species are defined in Eq. 6 and 7, respectively. The Gibbs free energy of adsorption of the intermediate species at different Pt active sites are tabulated in Supplementary Table 5 and 6 for atop and bridge positions, respectively.

Supplementary Note 8. Reaction mechanism

The alkaline HER mechanism and the way to simulate its rate-limiting step are being controversially discussed with Volmer-Heyrovsky or Volmer-Tafel considered as the most likely mechanisms.[17] In addition, we also considered thermal or the chemical dissociation of water since it is related to the catalyst activity in water splitting. The Volmer step involves the breaking of the bonds in the water molecule to form one adsorbed H and one free OH^- species with an electron transfer involved. The adsorbed H species then participate in the subsequent Heyrovsky step for H_2 formation. The thermal water dissociation step is defined as the step to break water into adsorbed H and adsorbed OH species with no electron transfer.[18] It is necessary to define this step for the micro-kinetic modelling, as the model takes into consideration of the OH coverage, which has non-negligible effects on the binding properties of the catalyst. However, the rate of thermal water splitting is negligible compared to Pt-based HER, thus, we believe the Volmer step should be the dominant step for HER in our system. Note that we evaluated the reaction energies of Tafel and chemical water dissociation steps from binding energies of H and OH on two separated Pt_{140}/C_{60} models, that is, the co-adsorption effects were not considered. The acidic Volmer and acidic Heyrovsky steps were included in the microkinetic analysis to confirm the model's ability to correctly describe the transition between acidic and alkaline HER mechanisms.[19] The reaction energies of R_1, R_2, R_3 ,R_4 and R_6 include the applied electrode potential U in the equation as shown in Eq. 11, 12, 13, 14 and 16. The different possible reaction mechanisms and pertinent reaction steps are proposed below from R_1 to R_7.

R_1 Acidic Volmer: $H_3O^+ + Pt_x + e^- \rightarrow H/Pt_x + H_2O$

$$\Delta G_{R1}(U) = G(H/Pt_x) + G(H_2O) - G(H_3O^+) - G(Pt_x) + |q_e|U \quad [11]$$

R_2 Acidic Heyrovsky: $H_3O^+ + H/Pt_x + e^- \rightarrow H_2 + H_2O + Pt_x$

$$\Delta G_{R2}(U) = G(Pt_x) + G(H_2) + G(H_2O) - G(H_3O^+) - G(H/Pt_x) + |q_e|U \quad [12]$$

R_3 Alkaline Volmer: $H_2O + Pt_x + e^- \rightarrow H/Pt_x + OH^-$

$$\Delta G_{R3}(U) = G(H/Pt_x) + G_{eff}(OH) - G(H_2O) - G(Pt_x) + |q_e|U \quad [13]$$

R_4 Alkaline Heyrovsky: $H_2O + H/Pt_x + e^- \rightarrow H_2 + OH^- + Pt_x$

$$\Delta G_{R4}(U) = G(Pt_x) + G_{eff}(OH) + G(H_2) - G(H_2O) - G(H/Pt_x) + |q_e|U \quad [14]$$

R_5 Alkaline Tafel: $(H + H)/Pt_x \rightarrow H_2 + Pt_x$

$$\Delta G_{R5} = 2 \times G(Pt_x) + G(H_2) - 2 \times G(H/Pt_x) \quad [15]$$

R_6 OH adsorption: $OH^- + Pt_x \rightarrow OH/Pt_x + e^-$

$$\Delta G_{R6}(U) = G(OH/Pt_x) - G_{eff}(OH) - G(Pt_x) - |q_e|U \quad [16]$$

R_7 Chemical water dissociation: $H_2O + Pt_x \rightarrow (H + OH)/Pt_x$

$$\Delta G_{R7} = G(H/Pt_x) + G(OH/Pt_x) - G(H_2O) - 2 \times G(Pt_x) \quad [17]$$

Here we assume that H_3O^+ species are in equilibrium with H^+ species under acidic conditions

$$H_3O^+ \leftrightarrow H^+ + H_2O\ ;\ G(H_3O^+) = G(H^+) + G(H_2O)$$

The activation energies ΔG_a° for R_1, R_2, R_3, R_4, R_5, R_6, and R_7 under pH = 14 were estimated for Pt(111) in Ref. [19] to amount to 0.04 eV, 0.20 eV, 0.42 eV, 1.20 eV, 0.72 eV, and 1.02 eV, respectively. We used these values to estimate the activation energies of R1-R7 on various sites of Pt/C_{60}(011) as

$$\Delta G_a = \Delta G_a^{\circ} + \Delta G_r[Pt_x] - \Delta G_r[Pt(111)] \quad [18]$$

Where $\Delta G_r[Pt_x]$ and $\Delta G_r[Pt(111)]$ are the reaction Gibbs energies of the steps under consideration calculated in this work on Pt/C_{60}(011) or nanocomposite or unsupported Pt and Pt(111) surface, respectively. The reaction rate constants for every step were calculated as

$$\text{Rate Constant of } R_i\text{: } k_i = \frac{k_B T}{h} e^{\left(\frac{-\Delta G_{a_i}}{k_B T}\right)} \quad [19]$$

where ΔG_{a_i} is the extrapolated activation energy of the reaction step, k_B is the Boltzmann constant, T is the temperature, and h is the Planck's constant.

The ΔG values for acidic and alkaline Volmer, Heyrovsky, Tafel and thermal water

dissociation steps are tabulated in Supplementary Table 7-8 for atop and bridge positions respectively. The activation energies differ significantly across the different interface Pt atoms in the supported Pt/C_{60} generating the sites with exponentially high and exponentially low activities in various reaction steps as explained in the microkinetic analysis below.

The rate constants of various alkaline steps were calculated at 0 V vs RHE for the forward reaction to compare how the reaction rates differ across different Pt sites on the interface of Pt/C_{60} as compared to unsupported Pt NCs and Pt nanoparticles supported on graphene. There are 7 corner Pt atoms, 6 edge Pt atoms, and 4 terrace Pt atoms bonded to fullerene molecules in Pt/C_{60}, whereas the interface of Pt nanoparticles with graphene contains 4 corner atoms and 8 edge atoms. For consistency, the current density calculated on Pt/C_{60}, Pt/graphene and unsupported Pt nanoparticles is normalized to the same amount of surface sites. The forward reaction rate constants shed light on the enhancement of the alkaline HER rates in the presence of the C_{60} support. Specifically, the Volmer step is enhanced by 94 and 90 times, the Heyrovsky step by 21 and 15 times, the Tafel step by 94 and 90 times, and the thermal water dissociation by 9 and 8 times for the 17 interfacial Pt-sites on Pt/C_{60} as compared to the unsupported Pt NCs and the interfacial Pt-sites on Pt/graphene (Supplementary Table 9), respectively. The bridge Pt positions also exhibit much higher reaction rate constants for all reaction steps (Supplementary Table 10). Supplementary Fig. 39 shows a huge diversity of the reaction rates on various sites on the Pt|C_{60} interface leading to the overall enhancement of HER activity of Pt/C_{60} catalysts compared to unsupported Pt particles and Pt nanoparticle on graphene support.

The microkinetic model for the systems was calculated using the rate constant (k) of each reaction calculated as follows:

$$r_1 = k_{1+} C_{H3O+}\, \theta_* - k_{1-} C_{H2O}\, \theta_H \qquad [20]$$

$$r_2 = k_{2+}C_{H3O+}\,\theta_H - k_{2-}C_{H2O}P_{H2}\,\theta_* \quad [21]$$

$$r_3 = k_{3+}C_{H2O}\,\theta_* - k_{3-}C_{OH-}\,\theta_H \quad [22]$$

$$r_4 = k_{4+}C_{H2O}\,\theta_H - k_{4-}C_{OH-}P_{H2}\,\theta_* \quad [23]$$

$$r_5 = k_{5+}\theta_H^2 - k_{5-}P_{H2}\theta_*^2 \quad [24]$$

$$r_6 = k_{6+}C_{OH}\,\theta_* - k_{6-}\,\theta_{OH} \quad [25]$$

$$r_7 = k_{7+}C_{H2O}\theta_*^2 - k_{7-}\theta_{OH}\theta_H \quad [26]$$

The r_i, k_{i+}, and k_{i-} represent the reaction rate, forward rate constants, and reverse rate constants for the different reactions of R_1 to R_7 respectively. The C_{H3O+}, C_{H2O}, and C_{OH} represent the concentrations of H^+ ions, H_2O, and OH ions respectively. The θ_*, θ_H, and θ_{OH} represent the free active sites and active sites with an H and OH adsorbed respectively, the total number of sites can be normalized:

$$\theta_* + \theta_H + \theta_{OH} = 1 \quad [27]$$

The number of free active sites and active sites with an H and OH adsorbed is constant in the steady state, which leads to the following equations:

$$\frac{d\theta_*}{dt} = r_1 - r_2 + r_3 - r_4 - 2r_5 + r_6 + 2r_7 = 0 \quad [28]$$

$$\frac{d\theta_H}{dt} = r_1 - r_2 + r_3 - r_4 - 2r_5 + r_7 = 0 \quad [29]$$

$$\frac{d\theta_{OH}}{dt} = r_6 + r_7 = 0 \quad [30]$$

The system of differential equations is solved with the introduction of initial parameters such as the concentrations of H_3O^+, OH^-, and H_2O together with the pressure of H_2 gas and the applied potential, which is varied from 0 to -0.40 V vs RHE to achieve the current density of 10 mA/cm^2 for unsupported Pt NCs. The ΔG_a and ΔG_R are adjusted accordingly in the presence of applied potential as shown in Supplementary Fig. 3 of Ref.14. The symmetry factor, β is assumed to be 0.5 with a symmetrical energy barrier which is an assumption commonly utilized in electrochemistry theoretical studies. The local electrode current passing through each site can be calculated from the rates of

steps 1, 2, 3, 4 and 6. The electron transfer rate is converted to current density for one Pt site. The reaction rates of r_2, r_4, and r_5 are summed up to calculate the rate of H_2 production. Based on these equations, we calculated the rate of H_2 production and current density as a function of the electrode potential (V vs RHE) for pH = 14 used in the experiments (Supplementary Fig. 4 in the main text). The microkinetic analysis shows that there is a significant enhancement of around 22 times in the current density for interface sites on supported Pt/C_{60} (011) as compared to unsupported Pt NCs for electrode potential up to –0.40 V. There is a smaller enhancement of around 8 times in the current density for Pt sites on Pt/graphene as compared to unsupported Pt NCs for electrode potential up to -0.40 V.

It is important to note that Pt atoms that are not in contact with the fullerene molecules will also contribute to the HER activity of Pt/C_{60} catalysts. If we consider all 96 Pt atoms on the surface of the Pt/C_{60}(011) model in microkinetic analysis, the overpotential required to achieve the current density of 10 mA cm^{-2} increases to −0.3 V, which is 0.09 V lower than the overpotential for unsupported Pt particle (Supplementary Fig. 41). Such change in overpotential results in ~5 times higher current density on Pt/C_{60}(011) model compared to unsupported Pt particle at –0.3 V vs RHE electrode potential. Similar results are observed when all the surface Pt sites are considered on Pt/graphene, yielding 0.08 V higher overpotential compared to the activity of the averaged Pt sites of Pt/C_{60}(011) at the current density of 10 mA cm^{-2}.

Note that the proposed HER mechanisms imply that H atoms are the only important intermediate species adsorbed on the catalyst surface in the alkaline HER reaction. The diffusion barriers for H atoms between different sites on Pt nanoparticles were calculated to be up to 0.22 eV on nanoparticles edges (Supplementary Fig. 40), whereas previous studies suggest even lower barriers for H diffusion on extended Pt surfaces.[20] Thus, the rate of H diffusion on Pt nanoparticles greatly exceeds the rate of HER, which

implies that adsorbed H atoms can easily migrate between the surface sites during the reaction. For example, H atoms could migrate from the sites that are highly active in Volmer step to the sites that are highly active in Tafel or Heyrovsky steps, which would enhance the effect of the diverse binding properties on Pt/C_{60} interfaces on the HER rate.

Supplementary Note 9. Efficiency of AEM electrolyser:

$TOF_{hydrogen}$ of AEM electrolyser at 1A cm^{-2}:

$TOF_{hydrogen}$ = 1 A cm^{-2} s/Q_{H2} = $6.24\times10^{18}/2$ s^{-1} cm^{-2}

= 3.12×10^{18} s^{-1} cm^{-2}

Caloric power of producted hydrogen fuel and the AEM electrolyser at 1A cm^{-2}:

$P_{hydrogen}$ = $LHV_{H2}\times(TOF_{hydrogen}/N_A)$=286 kJ mol^{-1} × (3.12×10^{18} s^{-1}/6.02e^{23}) cm^{-2}

=1.48 W cm^{-2}

$P_{AEM} = I \times E_{working\ voltage}$

Energy efficiency of the AEM electrolyser at 1A cm^{-2}:

$E = P_{hydrogen}/P_{AEM}$

Energy efficiency based on PtC_{60} cathode catalysts at 1 A cm^{-2} = $P_{hydrogen}/P_{AEM\text{-}Pt}C_{60}$

= 1.48 W/ 2.01 W = 74.0%

Energy efficiency based on Pt/C cathode catalysts at 1 A cm^{-2} = $P_{hydrogen}/P_{AEM\text{-}Pt/C}$

= 1.48 W/ 2.18 W = 67.8%

Energy efficiency based on Pt NCs cathode catalysts at 1 A cm^{-2} = $P_{hydrogen}/P_{AEM\text{-}Pt}C_{60}$

= 1.48 W/ 2.58 W = 57.3%

$TOF_{hydrogen}$ is turnover frequency of hydrogen molecule, $P_{hydrogen}$ is caloric power of synthesized H_2 of AEM, P_{AEM} is power of AEM, LHV_{H2} is lower heating value of hydrogen of 286 kJ mol^{-1}.

Reference

1. Ledezma-Yanez, I. *et al.* Interfacial water reorganization as a pH-dependent descriptor of the hydrogen evolution rate on platinum electrodes. *Nat. Energy* **2**, 17031 (2017).
2. Barber, J. H. & Conway, B. E. Structural specificity of the kinetics of the hydrogen evolution reaction on the low-index surfaces of Pt single-crystal electrodes in 0.5 M dm−3 NaOH1Dedicated to Professor W. Vielstich on the occasion of his 75th birthday.1. *J. Electroanal. Chem.* **461**, 80–89 (1999).
3. Lin, D. & Lasia, A. Electrochemical impedance study of the kinetics of hydrogen evolution at a rough palladium electrode in acidic solution. *J. Electroanal. Chem.* **785**, 190–195 (2017).
4. Xu, W., Lu, Z., Sun, X., Jiang, L. & Duan, X. Superwetting Electrodes for Gas-Involving Electrocatalysis. *Acc. Chem. Res.* **51**, 1590–1598 (2018).
5. Chahine, G. L., Kapahi, A., Choi, J.-K. & Hsiao, C.-T. Modeling of surface cleaning by cavitation bubble dynamics and collapse. *Ultrason. Sonochem.* **29**, 528–549 (2016).
6. Teran, L. A., Laín, S., Jung, S. & Rodríguez, S. A. Surface damage caused by the interaction of particles and a spark-generated bubble near a solid wall. *Wear* **438–439**, 203076 (2019).
7. Zheng, J., Yan, Y. & Xu, B. Correcting the Hydrogen Diffusion Limitation in Rotating Disk Electrode Measurements of Hydrogen Evolution Reaction Kinetics. *J. Electrochem. Soc.* **162**, F1470–F1481 (2015).
8. Hansen, J. N. *et al.* Is There Anything Better than Pt for HER? *ACS Energy Lett.* **6**, 1175–1180 (2021).
9. Sheng, W., Gasteiger, H. A. & Shao-Horn, Y. Hydrogen Oxidation and Evolution Reaction Kinetics on Platinum: Acid vs Alkaline Electrolytes. *J. Electrochem. Soc.* **157**, B1529 (2010).
10. Heiney, P. A. *et al.* Orientational ordering transition in solid C_{60}. *Phys. Rev. Lett.* **66**, 2911–2914 (1991).
11. Jeong, G.-U. & Lee, B.-J. Interatomic potentials for Pt-C and Pd-C systems and a study of structure-adsorption relationship in large Pt/graphene system. *Comput. Mater. Sci.* **185**, 109946 (2020).
12. Thompson, A. P. *et al.* LAMMPS - a flexible simulation tool for particle-based materials modeling at the atomic, meso, and continuum scales. *Comput. Phys. Commun.* **271**, 108171 (2022).
13. Kresse, G. & Furthmüller, J. Efficient iterative schemes for ab initio total-energy calculations using a plane-wave basis set. *Phys. Rev. B* **54**, 11169–11186 (1996).
14. Kresse, G. & Joubert, D. From ultrasoft pseudopotentials to the projector augmented-wave method. *Phys. Rev. B* **59**, 1758–1775 (1999).
15. Perdew, J. P., Burke, K. & Ernzerhof, M. Generalized Gradient Approximation Made Simple. *Phys. Rev. Lett.* **77**, 3865–3868 (1996).
16. Grimme, S., Ehrlich, S. & Goerigk, L. Effect of the damping function in dispersion corrected density functional theory. *J. Comput. Chem.* **32**, 1456–1465 (2011).

17. Jin, H. *et al.* Emerging Two-Dimensional Nanomaterials for Electrocatalysis. *Chem. Rev.* **118**, 6337–6408 (2018).
18. Wang, J. *et al.* Manipulating the Water Dissociation Electrocatalytic Sites of Bimetallic Nickel-Based Alloys for Highly Efficient Alkaline Hydrogen Evolution. *Angew. Chemie Int. Ed.* **61**, e202202518 (2022).
19. Lamoureux, P. S., Singh, A. R. & Chan, K. pH Effects on Hydrogen Evolution and Oxidation over Pt(111): Insights from First-Principles. *ACS Catal.* **9**, 6194–6201 (2019).
20. Fearon, J. & Watson, G. W. Hydrogen adsorption and diffusion on Pt {111} and PtSn {111}. *J. Mater. Chem.* **16**, 1989–1996 (2006).
